# Transcriptome Analysis of Polyhydroxybutyrate Cycle Mutants Reveals Discrete Loci Connecting Nitrogen Utilization and Carbon Storage in *Sinorhizobium meliloti*

**DOI:** 10.1128/mSystems.00035-17

**Published:** 2017-09-12

**Authors:** Maya D’Alessio, Ricardo Nordeste, Andrew C. Doxey, Trevor C. Charles

**Affiliations:** Department of Biology, University of Waterloo, Waterloo, Ontario, Canada; Rice University

**Keywords:** RNA-seq, *Sinorhizobium meliloti*, carbon nitrogen balance, carbon storage, glycogen, polyhydroxybutyrate (PHB)

## Abstract

Polyhydroxybutyrate (PHB) and glycogen polymers are produced by bacteria as carbon storage compounds under unbalanced growth conditions. To gain insights into the transcriptional mechanisms controlling carbon storage in *Sinorhizobium meliloti*, we investigated the global transcriptomic response to the genetic disruption of key genes in PHB synthesis and degradation and in glycogen synthesis. Under both nitrogen-limited and balanced growth conditions, transcriptomic analysis was performed with genetic mutants deficient in PHB synthesis (*phbA*, *phbB*, *phbAB*, and *phbC*), PHB degradation (*bdhA*, *phaZ*, and *acsA2*), and glycogen synthesis (*glgA1*). Three distinct genomic regions of the pSymA megaplasmid exhibited altered expression in the wild type and the PHB cycle mutants that was not seen in the glycogen synthesis mutant. An Fnr family transcriptional motif was identified in the upstream regions of a cluster of genes showing similar transcriptional patterns across the mutants. This motif was found at the highest density in the genomic regions with the strongest transcriptional effect, and the presence of this motif upstream of genes in these regions was significantly correlated with decreased transcript abundance. Analysis of the genes in the pSymA regions revealed that they contain a genomic overrepresentation of Fnr family transcription factor-encoding genes. We hypothesize that these loci, containing mostly nitrogen utilization, denitrification, and nitrogen fixation genes, are regulated in response to the intracellular carbon/nitrogen balance. These results indicate a transcriptional regulatory association between intracellular carbon levels (mediated through the functionality of the PHB cycle) and the expression of nitrogen metabolism genes.

**IMPORTANCE** The ability of bacteria to store carbon and energy as intracellular polymers uncouples cell growth and replication from nutrient uptake and provides flexibility in the use of resources as they are available to the cell. The impact of carbon storage on cellular metabolism would be reflected in global transcription patterns. By investigating the transcriptomic effects of genetically disrupting genes involved in the PHB carbon storage cycle, we revealed a relationship between intracellular carbon storage and nitrogen metabolism. This work demonstrates the utility of combining transcriptome sequencing with metabolic pathway mutations for identifying underlying gene regulatory mechanisms.

**Author Video**: An author video summary of this article is available.

## Abstract (precis)

The ability of bacteria to store carbon and energy as intracellular polymers uncouples cell growth and replication from nutrient uptake and provides flexibility in the use of resources as they are available to the cell. The impact of carbon storage on cellular metabolism would be reflected in global transcription patterns. By investigating the transcriptomic effects of genetically disrupting genes involved in the PHB carbon storage cycle, we revealed a relationship between intracellular carbon storage and nitrogen metabolism. This work demonstrates the utility of combining transcriptome sequencing with metabolic pathway mutations for identifying underlying gene regulatory mechanisms.

## INTRODUCTION

Symbiotic nitrogen-fixing nodules form on alfalfa roots as the result of a partnership between the plant and *Sinorhizobium meliloti* bacteria (1). Prior to infection, bacterial cells within the rhizosphere receive specific signals from the susceptible host in the form of flavonoid molecules such as luteolin. In response to these signals, *nod* genes are induced, which results in the production of lipochito-oligosaccharide molecules that signal the initiation of root nodule formation. This leads to the critical process of infection of the nascent root nodule and, in turn, the formation of differentiated bacteroids that are able to fix nitrogen, an energy-demanding process that is fueled by photosynthate-derived dicarboxylic acids (2).

The transition from rhizosphere colonization through to infection and then differentiation to a mature bacteroid is central to the nodule developmental pathway. Mutant analysis has identified several contributing factors, including extracytoplasmic molecules such as succinoglycan, galactoglucan, and lipopolysaccharide, and these could be involved in plant-bacterium recognition, which guides the infection process. Alfalfa root exudates contain a variety of compounds, some of which are host specific and act as signals to *S. meliloti* in the soil (3). Successful chemotaxis is essential for cells to be competitive for nodulation (4).

Besides recognition, an important consideration is the carbon and energy requirements of the infection process, in which the infecting bacterial cells progress through the infection thread by cell division (5). It has been hypothesized that this growth in the infection thread is fueled by intracellular carbon stores, primarily polyhydroxybutyrate (PHB), that accumulate in the nutrient-rich rhizosphere environment (6). *S. meliloti* cells within the infection thread tend to contain PHB granules, but once the cells differentiate into bacteroids, the PHB granules are no longer present (7, 8). Although mutants unable to synthesize PHB are able to infect the host, leading to nitrogen fixation, they are severely deficient in infection ability when in direct competition with strains that are able to synthesize PHB (9). Thus, PHB synthesis appears to play an important role in symbiosis initiation although not a required role in a laboratory environment.

**FIG 1:**
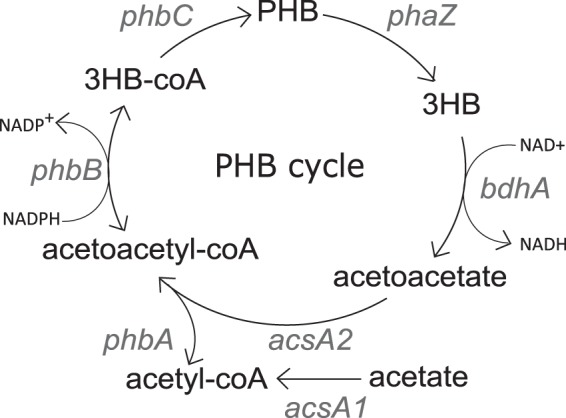
Overview of the synthesis and degradation of PHB in *S. meliloti*. Carbon enters the pathway in the form of acetyl-CoA, which is converted to PHB and then degraded back to acetyl-CoA.

The genetics of the PHB synthesis and degradation pathways have been thoroughly characterized in *S. meliloti* (Fig. 1). Acetoacetyl coenzyme A (acetoacetyl-CoA) is formed via the condensation of two acetyl-CoA molecules, catalyzed by *phbA*-encoded acetoacetyl-CoA thiolase (EC 2.3.1.9) (10). The acetoacetyl-CoA is then reduced to 3-hydroxybutyryl-CoA by *phbB*-encoded acetoacetyl-CoA reductase (EC 1.1.1.36) (11). The resulting 3-hydroxybutyryl-CoA is polymerized by *phbC*-encoded PHB polymerase (EC 2.3.1.B2) (12). Intracellular PHB is degraded by *phaZ*-encoded depolymerase (EC 3.1.1.75) to d-3-hydroxybutyrate, which is then oxidized by d-3-hydroxybutyrate dehydrogenase to acetoacetate by the short-chain dehydrogenase/reductase d-3-hydroxybutyrate dehydrogenase (EC 1.1.1.30) (8, 13). The *acsA2*-encoded acetoacetyl-CoA synthetase (EC 6.2.1.16) then catalyzes the activation of acetoacetate to acetoacetyl-CoA for use in the pathway again (14). *phbC* mutants have been shown to be unable to produce detectable amounts of PHB. A *phaZ* mutant was shown to accumulate more PHB than the wild type, while a *glgA1* mutant showed less PHB accumulation than the wild type (15, 16).

While the regulatory mechanisms that govern PHB accumulation remain unclear, evidence suggests that the PHB cycle is linked to the regulation of other carbon storage mechanisms (exopolysaccharide [EPS] and glycogen production), as well motility and symbiosis. For example, a *phbC* mutation has been shown to result in decreased EPS levels (succinoglycan [EPSI] and galactoglucan [EPSII]) and increased glycogen levels, as well as a decreased ability to compete for nodule infection (9, 17). Mutations in *chvI* lead to hypermotility and altered PHB accumulation (18).

Glycogen is produced in *S. meliloti* as a reserve carbon store, similar to PHB (15). There is not a clear mechanism for the allocation of carbon between glycogen synthesis and PHB synthesis in *S. meliloti*. In cyanobacteria, it has been proposed that carbon is diverted to glycogen (over PHB) during nitrogen stress (19). Unlike PHB, glycogen forms granules that are water soluble, and they are generally much smaller than PHB granules (20). Glycogen is synthesized through the production of ADP-glucose by pyrophosphorylase, GlgP, followed by the sequential addition of these molecules to a growing α-1,4-glucan chain by glycogen synthase (GlgA1) and glycogen branching enzyme (GlgB1) (21). Glycogen can be debranched to its individual units by the glycogen-debranching enzyme GlgX1 to free up carbon during times of metabolic activity. Disruption of the glycogen synthase gene *glgA1* results in decreased PHB levels and increased EPS levels compared to the wild type (15). Strains unable to synthesize glycogen are able to form nodules and fix nitrogen, but they exhibit a noticeable delay in nodulation and a reduction in competitive ability (15).

Previous transcriptomics studies of *Ralstonia eutropha* (*Cupriavidus necator*) examined expression differences between the wild type (strain H16) and PHB synthesis mutants, as well as between growth phases. The PHB synthesis mutants exhibited increased expression of various PHB cycle and related genes (phasins), appeared to be defective in fatty acid metabolism, and showed downregulation of two *cbb* operons that are involved in CO_2_ assimilation (22). In another study, during PHB accumulation, repression of genes involved in central metabolism was observed in addition to active transcription of *cbb* and β-oxidation genes (23). This work also showed consistently high transcription of the PHB cycle-associated genes *phaR*, *phaC1*, and *phaB1* and some phasin genes (23).

Previous studies have helped to establish knowledge of transcriptomic regulation in *S. meliloti*. Early work involved both macroarray and microarray investigations of symbiotic development, growth under micro-oxic conditions, acidic adaptation response, cell cycle and environmental signaling, Clr regulation, and the nitrogen stress response (24–32). More recently, transcriptome sequencing (RNA-seq) analysis has contributed to the general mapping of transcription start sites (TSS) and regulatory motifs. To our knowledge, the only specific application of RNA-seq in *S. meliloti* is to Hfq regulation (33, 34). Here, to investigate the influence of intracellular carbon storage mechanisms on global transcriptional regulation, we performed RNA-seq transcriptomic analyses of individual *S. meliloti* mutants in which each of the key reactions of the PHB cycle are disrupted. Given the role of glycogen as an intracellular carbon store, we also assessed a glycogen synthase gene (*glgA1*) mutant. We reasoned that by systematically examining the transcriptional effects of multiple PHB cycle disruptions, it might be possible to differentiate core regulatory determinants of the PHB pathway from pleotropic noise that might arise from single gene knockouts. In addition, by using a glycogen synthase mutant, we can differentiate the effects specific to PHB storage mutants from effects caused by mutation in any carbon storage system. By comparing the transcriptomic profiles of the different mutants, we identified core loci of differential transcriptional activity that reside on the pSymA megaplasmid and contain numerous genes involved in nitrogen utilization, nitrogen fixation, and denitrification. Analysis of these genes and their regulatory regions suggests a relationship between intracellular carbon accumulation and the regulation of nitrogen metabolism genes. We are thus continuing the use of RNA-seq technology to delve more deeply into specific regulatory pathways in *S. meliloti*.

## RESULTS AND DISCUSSION

### Rm1021 shows large-scale significant differences in the transcription of carbon, nitrogen, and symbiosis-associated genes under nitrogen-limited conditions. (i) Establishment of conditions for differential PHB accumulation.

It has been established that *S. meliloti* accumulates substantial amounts of PHB during growth on YM medium, which is carbon rich due to high levels of mannitol and contains limiting nitrogen (16). We first determined that increasing the amount of yeast extract 20-fold resulted in the absence of detectable PHB accumulation, likely because of relief of the unbalanced carbon/nitrogen ratio. We therefore adopted the original and yeast extract-enhanced versions of YM medium as PHB-accumulating and -nonaccumulating medium conditions and termed these conditions nitrogen limited and balanced, respectively, in line with the ratio of carbon and nitrogen levels. Nitrogen-limited conditions consistently resulted in the wild-type strain accumulating substantial PHB at stationary phase, whereas balanced conditions always resulted in undetectable PHB levels. Glycogen is also known to be produced in YMB medium, which is the base medium that was modified for the nitrogen-limited conditions in this work (15).

Gene expression profiles were determined at mid to late log phase under both conditions (see Fig. S1A in the supplemental material). Comparison of the gene expression profiles of parental strain Rm1021 under these two conditions revealed within-strain (wild type to wild type) differential expression of 2,335 genes in total, with 1,096 that were upregulated under nitrogen-limited conditions, showing increased transcript abundance, and 1,239 that were downregulated. It should be cautioned, however, that this effect is not likely to be solely related to carbon storage, as the differences in the yeast extract concentration likely influenced numerous metabolic pathways.

10.1128/mSystems.00035-17.1 FIG S1  (A) Growth curves of wild-type Rm1021 and mutants under nitrogen-limited (right) and balanced (left) conditions. Values below 0.1 are not shown, as the read error is high at low growth because of the presence of precipitates in the growth medium. (B) Venn diagram of genes determined to be significantly different in the *cyaJ* overexpression transcriptome, the phosphate starvation transcriptome, and the Rm1021 nitrogen-limited transcriptome in this study (32, 57). (C) Frequency of transcripts over a log_2_-fold change binned distribution in all mutants under both balanced (light blue) and nitrogen-limited (dark blue) conditions. (D) The 120-kb region showing the locations of the motifs and genes in the region, with experimental TSS predictions added (33). Download FIG S1, EPS file, 1.3 MB. Copyright © 2017 D’Alessio et al. 2017 D’Alessio et al. This content is distributed under the terms of the Creative Commons Attribution 4.0 International license.

**FIG 2:**
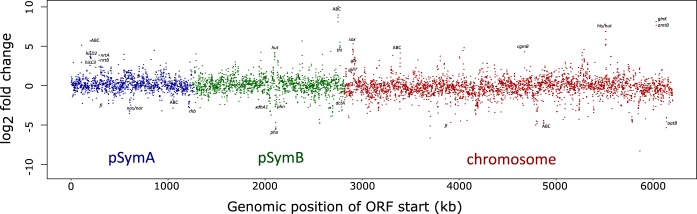
Within-strain differential expression (log_2_-fold change) of each gene in *S. meliloti* Rm1021 under nitrogen-limited conditions compared to balanced conditions, mapped across the genome. Individual genes and operons of interest are indicated, and hypothetical ABC transporters are identified as ABC.

An overview of the differential expression in Rm1021 under balanced and nitrogen-limited conditions across the coordinates of the genome (Fig. 2) demonstrates an even distribution of effect across the three replicons, the megaplasmid pSymA, the chromid pSymB, and the chromosome. Key genes of interest are indicated and discussed in detail below.

### (ii) Differentially expressed carbon storage genes.

Of note, the acetoacetyl-CoA synthetase-encoding gene *acsA2* (accession no. SMc00774; see below), was the only PHB cycle gene differentially expressed under the two conditions. Hence, the PHB cycle genes are not transcriptionally regulated in response to environmental changes that affect PHB accumulation, thus implying that regulation of PHB cycle activity and PHB accumulation occurs at the translational or posttranslational level. We found that two glycogen synthesis gene transcripts, *glgA1* and *glgX1*, differed between the conditions, with increased transcript abundance under nitrogen-limited conditions, indicating that they may be transcriptionally regulated in response to the extracellular carbon and/or nitrogen supply.

The downregulation of the *acsA2*-encoded acetoacetyl-CoA synthetase raises the possibility that this enzyme represents a key control point in the PHB cycle. However, since *acsA2* mutants are not known to hyperaccumulate PHB, it may alternatively reflect the lower levels of available acetoacetate substrate, considering that flux through the degradation pathway would be reduced under these conditions. Why this would be different for *acsA2* than other PHB cycle genes is not known and bears further investigation.

The two phasin genes *phaP1* (accession no. SMc00777) and *phaP2* (accession no. SMc02111) were upregulated under nitrogen-limited conditions, while there was no significant difference in *aniA* (accession no. SMc03880), *phaR* in other species, under the two conditions. This was not unexpected, as PhaP1 and PhaP2 are well established as key proteins in the formation of PHB granules (17). In *R. eutropha*, phasin genes were also found to be upregulated in response to mutation of the PHB synthesis genes (22). Previous studies have implicated *aniA* in the regulation of PHB and EPS synthesis under microaerobic conditions (35). The lack of difference in *aniA* transcription suggests that AniA’s activity is not transcriptionally regulated, as AniA has been shown to repress the transcription of phasin genes in several species, although this has not been directly shown in *S. meliloti*, and here the phasin genes are upregulated (36–38). Insertional inactivation of *aniA* in *S. meliloti* Rm41 results in decreased PHB accumulation, demonstrating a role in the regulation of PHB production (35). Recently, PhaR in *Bradyrhizobium diazoefficiens* was shown to be a global regulator of excess carbon allocation and symbiosis through control of the *fixK2* gene (37). The increase in phasin gene expression under nitrogen-limited conditions observed here could be mediated by AniA or another transcriptional regulator.

### (iii) Differentially expressed nitrogen-associated genes.

The most strongly upregulated genes under nitrogen-limited conditions were those involved in amino acid and nitrogen uptake and utilization. This most certainly reflects the very different availability of nitrogenous compounds under the two conditions because of the yeast extract concentration differences. Included among the genes with the strongest transcript level upregulation were several that encode amino acid uptake and binding proteins, as well as nitrate uptake genes. The amino acid uptake genes include the *aap* operon (SMc02118 to SMc02121) and the *liv* (*bra*) operon (SMc01946 to SMc01951), both involved in branched-chain amino acid uptake, as well as the *hut* (SMb21163 to SMb21166) and *his* (SMc00669 to SMc00674) operons, involved in histidine and proline uptake (39, 40). These transcriptional changes are all consistent with transcriptional regulation (induction or removal of repression) of amino acid uptake genes in response to the scarcity of nitrogenous compounds. Interestingly, this finding has similarities to another study that detected significant differences in the transcription of amino acid uptake genes in a *cyaJ* overexpression mutant of *S. meliloti* (32) (further discussed below). Under nitrogen-limited conditions, exogenous levels of amino acids are expected to be much lower than under balanced conditions, which would influence the expression of genes involved in the uptake and utilization of amino acids.

Studies have demonstrated that when *S. meliloti* is grown under nitrogen-limited conditions, characterized by a low glutamine/α-ketoglutarate ratio, the cell activates the nitrogen stress response, mediated by GlnD through GlnK and GlnB (41). Activated GlnK and GlnB contribute to the activation of NtrC to increase the catabolism of nitrogen-containing compounds, freeing up ammonium. Both *glnK* and *glnB* (SMc03806 and SMc00947) were upregulated under nitrogen-limited conditions, while *ntrC* and *glnD* were not. *glnK* was the fourth most upregulated transcript under nitrogen-limited conditions, suggesting that it could be a key component of the observed cellular response. This finding suggests that the nitrogen stress response is transcriptionally regulated through the upregulation of *glnK* and *glnB*, whose protein products then trigger catabolism (through the activation of NtrC) of intracellular nitrogenous compounds to free nitrogen stores in the cell. Previous work with *Azospirillum brasilense* has implicated NtrB and NtrC in the regulation of PHB synthesis in response to the ammonia concentration in the medium (42). Consistent with the activation of NtrC, we observed upregulation of the following known transcriptional targets of NtrC: *glnII*, which encodes glutamine synthetase (GS; SMb20745), *glnE* (SMc02368), which encodes an adenylyltransferase that inactivates GS, and *amtB* (SMc03807), which encodes an ammonium transporter.

The *glx* operon (SMc02610 to SMc02612), which codes for glutamate synthase, was also upregulated, while *glxA* (SMc02609), the regulator of the operon, was downregulated. This suggests that *glxA* acts as an inhibitor of *glx* transcription. Immediately upstream of the *glx* operon is the *sox* operon (*soxGA1BD*), which was starkly upregulated under nitrogen-limited conditions. The *sox* operon encodes sarcosine oxidase, which degrades sarcosine, an intermediate in glycine synthesis and degradation (43).

The GSIII-encoding gene *glnT* (SMc02613) was also upregulated, although it had previously been reported to be expressed only when GSI and GSII are absent (44). The expression of GSI-encoding *glnA* did not change under the two conditions, and out of three putative GSs, one showed no difference (SMc00762), one was upregulated (SMc01594), and the other was downregulated (SMc02352). This suggests areas in which experimental investigation would be fruitful, as these results are not consistent with the existing body of literature or in some cases contradict previous findings, as discussed above.

### (iv) Differentially expressed symbiosis-associated genes.

Cyclic β-1,2-glucan is a symbiotically important cell surface polysaccharide. Transcript levels of the *ndvA* gene (SMc03900), which encodes a β-1,2-glucan export protein, were elevated under nitrogen-limited conditions. However, the *ndvB* gene, which encodes the transmembrane-located cyclic β-1,2-glucan production protein, was not differentially expressed. NdvA is responsible for the inner membrane transport of cyclic β-1,2-glucan and is essential for the establishment of an effective symbiosis (45). Our attention was drawn to cyclic β-1,2-glucan synthesis because of the strong upregulation of *cgmB* (SMc04438). CgmB is involved in the modification of cyclic β-1,2-glucan after synthesis, which is critical during infection (46).

The *dctA* gene (SMb20611), located on the chromid, showed a strong decrease in transcription under nitrogen-limited conditions. DctA is a dicarboxylic acid transporter used by the cell during symbiosis to transport in dicarboxylic acids, such as malate, provided by the host plant. This downregulation may be a response to high intracellular carbon levels, as further import of organic acids into the cell would increase the intracellular carbon content. It is possible that this is not specific to *dctA* but instead is due to a global repression effect on multiple carbon uptake pathways, even those that would normally be inactive.

EPSs are critically important to the interaction of *S. meliloti* with its plant host, leading to the development and infection of nitrogen-fixing root nodules. The synthesis of EPSs has long been associated with carbon-sufficient conditions under which growth is hindered by low levels of other essential nutrients such as nitrogen (47). These conditions also promote PHB accumulation. In *S. meliloti* Rm1021, this is manifested in a mucoid colony morphology on agar plates (48). Consistent with this, we observed that several genes involved in the succinoglycan and galactoglucan EPS synthesis pathways were significantly upregulated under nitrogen-limited conditions. This includes many of the succinoglycan synthesis genes in the 35-kb region bounded by SMb20932 and *exoP* (SMb20961). Interestingly, the regulatory genes *exsF* (SMb20934), *exsI* (SMb20935), and *exsB* (SMb20940) were downregulated. Similarly, many of the genes in the 27-kb galactoglucan synthesis region between *wgeH* (SMb21307) and *wgaJ* (SMb21327) were significantly upregulated. This suggests that regulation of EPS accumulation is transcriptional and is in response to growth conditions. Although Rm1021 contains a null *expR* mutation, we observed upregulation of *sinI* (SMc00168) and *sinR* (SMc00170), which encode components of a quorum-sensing system that is known to positively regulate EPS synthesis (49, 50).

The operon containing *msbA2* (SMb21191) was observed to be significantly upregulated. MsbA2 has been suggested to be involved in EPS production and is essential for the establishment of a symbiosis between *S. meliloti* and alfalfa (51). Mutations in *msbA2* disrupt the infection leading to root nodule symbiosis, and the operon has also been shown to be regulated by the *exoR-exoS-chvI* regulatory system, which is known to also be required for succinoglycan and galactoglucan synthesis (28, 52). Interestingly, the expression of *exoS* (SMc04446) was significantly downregulated in Rm1021 under nitrogen-limited conditions.

### (v) Other metabolism.

Surprisingly, genes for phosphate and phosphonate uptake and utilization (SMb21174, SMb21175, SMb21176, and SMb2177) and purine metabolism (SMb21286, SMb21287, and SMb21288), were strongly downregulated. Rm1021 has a mutation in *pstC* that causes the cell to constantly respond as if it were in a state of phosphate stress via constitutive expression of the *pho* operon (53). Downregulation of the *pho* operon and purine metabolism genes is counterintuitive, as the extracellular phosphate and purine concentrations would be low in nitrogen-limited medium because of the decreased amount of yeast extract present. Also of note is the downregulation of many genes involved in the energy currency of the cell, such as those encoding components of the electron transport chain (SMb21488 and SMb21489), as well as those involved in core functions of transcription, translation, and homologous recombination (e.g., SMc00565, SMc01290, SMc01291, SMc02101, SMc01318, SMc01319, SMc01378, SMc01379, SMc03779, SMc01243, SMc00335, and SMc02692). This may reflect a decrease in central metabolism exhibited by the cell in response to nitrogen limitation.

The rhizobactin 1021 synthesis operon on pSymA (SMa2400 to SMa2410) was downregulated under nitrogen-limited conditions. Rhizobactin is a siderophore that captures iron for transport into the cell (54). This operon is known to be repressed under high-iron conditions, as might be expected under balanced conditions (55). The downstream genes *rhrA* and *rhtA* (SMa2412 and SMa2414) were similarly downregulated. RhtA is a known outer membrane receptor protein for rhizobactin 1021, while RhrA is a positive transcriptional regulator of *rhrA* and the rhizobactin operon (55). Both conditions have equal molarity of FeCl_3_ ⋅ 6H_2_O, but there is 20 times as much yeast extract, which is known to contain trace amounts of iron, under balanced conditions as under nitrogen-limited conditions. It is therefore surprising to observe decreased transcription of the rhizobactin synthesis operon under nitrogen-limited conditions. Previous studies provide an explanation for this phenomenon, as it has been suggested that the differential regulation of the gene for rhizobactin and other iron uptake genes is attributable to a general stress response (56, 57).

### (vi) Coregulation of carbon and nitrogen pathways.

Although none of the PHB cycle genes were upregulated under nitrogen-limited conditions, the *xdhA2* and *xdhB2* genes showed increased expression. Notably, these genes are involved in hypoxanthine degradation and are immediately adjacent to the *bdhA* gene, which encodes the key PHB degradation enzyme d-3-hydroxybutyrate dehydrogenase. In rhizosphere colonization and infection, it has been hypothesized that a regulatory association exists between PHB degradation and the degradation of nitrogen-containing metabolites (hypoxanthine) (58). Consistent with this idea, expression of the predicted LysR-type regulator (SMb20847) downstream of *xdhA2-xdhB2* was also upregulated, raising the possibility that it is somehow involved in the regulation of this operon. It was previously reported that SMb20847 in a *nifA* mutant background was upregulated compared to that in the wild-type background during symbiosis, which establishes this potential regulator’s involvement in symbiosis (26). Increased expression of these genes under nitrogen-limited conditions would contribute to increased breakdown of hypoxanthine in the cell to free up nitrogen for use in critical cell activities.

### (vii) Comparison to other *S. meliloti* transcriptomic data sets.

Previous studies of *S. meliloti* have generated transcriptomic data sets to assess gene expression under various conditions (24, 25, 31, 32, 57, 59–61). One previously generated transcriptome that may inform the results of this study examined global gene expression patterns following *cyaJ* overexpression (32). Specifically, in that study, the *cyaJ* gene was introduced on an expression plasmid and induced during log-phase growth, and 154 genes were detected as differentially expressed compared to the wild-type strain (32). Interestingly, in comparison with our data set, 86 of these were also differentially expressed in the parental strain under nitrogen-limited conditions compared to balanced conditions (Fig. S1B). The common genes include EPS (*exsI*, *exoF1*, *exoY*, *exoN*, *exoP*, *exoF3*, and *exoW*), flagella and chemotaxis (*flgB*, *flgC*, *glgG*, and *motB*), and respiration (*qxtA*, *qxtB*, *fbcF*, *fbcB*, *fbcC*, *ctaD*, *cycF*, *cyoC*, and *coaA*) genes, as well as *cyaJ* (Table S1).

10.1128/mSystems.00035-17.9 TABLE S1  Comparison of the *cyaJ* overexpression transcriptome, the phosphate starvation transcriptome, and the Rm1021 nitrogen-limited transcriptome. (A) Genes commonly determined to be significantly different in the *cyaJ* overexpression and Rm1021 nitrogen-limited transcriptomes. (B) Genes commonly determined to be significantly different in the phosphate starvation and Rm1021 nitrogen-limited transcriptomes. (C) Genes commonly determined to be significantly different in all three transcriptomes (32, 57). Download TABLE S1, XLSX file, 0.02 MB. Copyright © 2017 D’Alessio et al. 2017 D’Alessio et al. This content is distributed under the terms of the Creative Commons Attribution 4.0 International license.

Another previous study of interest examined the phosphate starvation response by comparing the transcriptomic profiles of Rm1021, Rm2011, and a *phoB* mutant under limited-phosphate conditions (57). That study identified 234 genes that were differentially expressed in the *phoB* mutant and wild-type strains (57). In comparison to our data set, 155 of these genes overlap those that were differentially expressed in the parental strain under nitrogen-limited conditions (Fig. S1B). The common genes include iron uptake genes (rhizobactin genes and others), EPS synthesis genes, xanthine dehydrogenase genes (*xdhA2* and *xdhB2*), phosphate and other ABC transporter genes, ribosomal protein genes, flagellar and chemotaxis protein genes, arginine transport genes, and 40 hypothetical genes.

Comparing the three transcriptomes collectively, only 17 genes were commonly differentially expressed (Fig. S1B). These genes include four EPS synthesis genes (*exoF1*, *exoY*, *exoN*, and *exoW*), a cytochrome gene (*cycF*), and two flagellar protein genes (*flgB* and *flgG*). It is difficult to interpret the relevance of this set of overlapping genes, which may speak more to the limited depth of microarray data than a lack of biological importance.

These results demonstrate that there are numerous differentially regulated genes under balanced versus nitrogen-limited conditions. Common themes include expression changes in genes related to nitrogen uptake and denitrification, nitrogen fixation, and nitrogen stress response and PHB-associated genes. This is in contrast with the observed lack of transcriptional change in genes directly associated with the two main carbon storage systems, PHB and glycogen. These findings demonstrate widespread transcriptional changes in nitrogen metabolism genes in the cell in response to a change in the environmental nitrogen profile.

### Genetic disruption of the PHB cycle leads to strong transcriptional downregulation of loci on pSymA. (i) PHB cycle mutants.

By applying transcriptomics to preconstructed *phbA*, *phbB*, *bdhA*, *acsA2*, and *glgA1* mutants, as well as newly generated *phbC*, *phbAB*, and *phaZ* mutants (see Materials and Methods), we analyzed global patterns of gene expression at multiple steps in the PHB pathway. We also included a glycogen synthase gene (*glgA1*) mutant as another carbon storage system mutant for comparison. For each mutant, we first confirmed the absence or reduction of the transcript corresponding to the mutated gene, according to the deletion or insertion nature of the mutation. For all of the genes, we then compared the transcript abundance in each mutant with that in the parental strain, which we refer to as between-strain differential expression. We are using this term to differentiate transcript abundance comparisons of the same strain under different conditions (within-strain differential expression) and comparisons between different strains under the same conditions, i.e., parental to mutant (between-strain differential expression). Earlier we discussed within-strain differential expression, the log_2_-fold change between balanced and nitrogen-limited conditions in the wild type. In this section, use of the term differential expression can be assumed to refer to the difference between strains.

**FIG 3:**
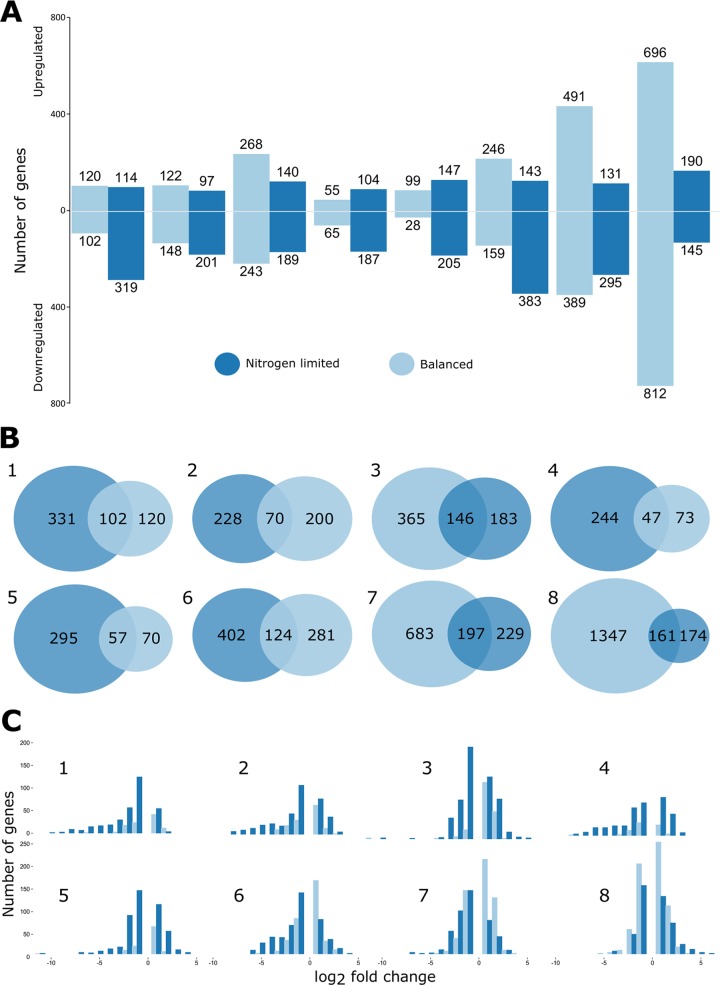
Initial analysis of the transcriptional effects in eight carbon storage mutants (1 to 8; Δ*phbA*, *phbB*::ΩSpSm, Δ*phbAB*, Δ*phbC*, Δ*phaZ*, Δ*bdhA*, *acsA2*::Tn*5*, *glgA1*ΔPstI) compared to the parental strain (Rm1021) under nitrogen-limited (dark blue) and balanced (light blue) conditions. (A) Numbers of genes differentially expressed (increased or decreased in abundance) from strains 1 to 8 left to right. (B) Overlap of different transcripts in each mutant between the two conditions. (C) Frequency of transcripts over a log_2_-fold change binned distribution of a greater-than-log_2_-fold change of 1 or a less-than-log_2_-fold change of −1.

When evaluating between-strain differential expression under each of the two conditions for each mutant, some general trends were recognized. The number of transcripts ranged from 120 (55 up/65 down) in the *phbC* mutant to >1,500 (696 up/812 down) in the *glgA1* mutant, both under balanced conditions (Fig. 3A). Interestingly, the *acsA2* and *glgA1* mutants both appeared to show larger numbers of differentially expressed genes under balanced conditions and a larger difference in the number of differentially expressed genes between the two conditions than the other mutants, which tended to be similarly affected by the two conditions. We directly examined the relationship between the differentially expressed transcripts in each mutant under the two conditions (Fig. 3B). For each mutant, the overlap of expression under the two conditions was relatively small. This underlines the relevance of examining transcriptional responses under the two different environmental conditions, only one of which results in PHB accumulation.

The genome-wide distribution of log_2_-fold changes in genes for each mutant and background was determined. There was consistency in the frequency distribution for each mutant between the conditions (Fig. S1C). However, when focusing on the genes showing the largest effect size (largest positive and negative log_2_-fold changes), substantial differences were observed in the mutants between conditions (Fig. 3C). Under nitrogen-limited conditions, a larger number of genes show extreme log_2_-fold values (in terms of both up- and downregulation). The most notable effect was the increase in genes showing negative log_2_-fold values. This trend excludes the *acsA2*, *glgA1*, and *bdhA* mutants, which still showed a high frequency of strong effect but less of a notable difference between the two conditions.

### (ii) Expression patterns in each of the mutant strains.

**FIG 4:**
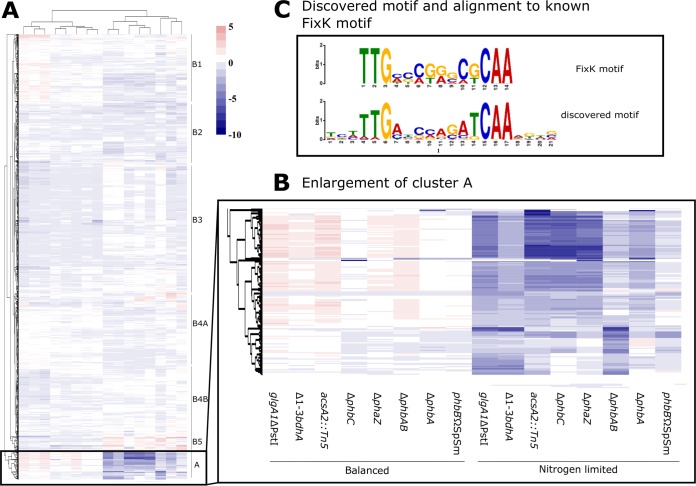
Clustering of mutants based on shared transcriptional patterns of expression used to perform *de novo* motif discovery resulting in a motif. (A) Hierarchically clustered heat map showing the between-strain differential expression (log_2_-fold change) of all transcripts that were significantly different in at least one mutant background under nitrogen-limited and balanced conditions. Clear clusters have been differentiated to allow for motif discovery analysis. (B) Enlargement of the transcriptional pattern in cluster A. (C) Discovered motif from cluster A aligned with the FixK motif.

The patterns of expression in the eight mutants were compared and visualized by using a clustered heat map (Fig. 4A; enlarged in Fig. S2). The clustering reflects the clear distinction between nitrogen-limited and balanced conditions. Under both conditions, the *acsA2* and *bdhA* degradation pathway mutants were tightly clustered together. Under balanced conditions, the *phbA*, *phbB*, and *phbAB* synthesis mutants were more closely associated with each other than with *phbC*. Surprisingly, the *phbC* and *phaZ* mutants had similar transcriptional patterns. The clustering of the *phbC* and *phaZ* mutants under balanced conditions possibly reflects their unique location in the PHB cycle, at the juncture between PHB synthesis and degradation.

10.1128/mSystems.00035-17.2 FIG S2  Hierarchically clustered heat map showing between-strain differential expression (log_2_-fold change) of all transcripts that were different in at least one mutant background under nitrogen-limited and balanced conditions. Clear clusters have been differentiated to allow for motif discovery analysis. Download FIG S2, EPS file, 2.3 MB. Copyright © 2017 D’Alessio et al. 2017 D’Alessio et al. This content is distributed under the terms of the Creative Commons Attribution 4.0 International license.

In contrast, under nitrogen-limited conditions, the *phbA*, *phbB*, and *phbC* mutants clustered together while the *phaZ* and *phbAB* mutants formed a loose cluster with the *glgA1* mutant. The discordant responses of the *phbA*, *phbB*, and *phbAB* mutants under nitrogen-limited conditions were unexpected, considering that *phbA* and *phbB* are organized in a single operon and the mutants were expected to behave similarly. The *phbA* and *phbAB* mutants would be expected to accumulate acetyl-CoA similarly, as the *phbA* mutation is nonpolar to *phbB*, but the resulting transcriptional differences do not correlate with this. A potential hypothesis could be that acetoacetyl-CoA is produced by other enzymes in the cell, acting as the substrate for the PhbB enzyme in the *phbA* mutant. This is unlikely, as the *phbA* mutant has been shown to be unable to produce detectable accumulation of PHB despite its deletion mutation being nonpolar on *phbB* (62).

This initial analysis of the RNA-seq data suggests important differences in the patterns of gene expression in the mutants under the two conditions.

### (iii) Commonly differentially expressed genes are overrepresented in distinct regions of pSymA.

To better visualize patterns of expression, the log_2_-fold changes in genes in each mutant background were mapped to their chromosomal locations and compared (Fig. S3 and S4). There was a clear distinction in the transcriptional response pattern of the mutants under nitrogen-limited conditions and that seen under balanced conditions. Strong common transcriptional changes were most evident under nitrogen-limited conditions in loci on pSymA (Fig. S3). Given the remarkable effect observed under nitrogen-limited conditions, we focused our attention on these common genes. The differentially expressed genes on pSymA are clustered in three distinct regions, the largest being a 120-kb segment bounded by SMa1077 and SMa1297, with 75% of the genes differentially expressed in at least one of the mutants. The two smaller regions, 37 and 7 kb, respectively, are separated from each other by 40 kb and bounded by SMa0631 and SMa0697 (50% of the genes differentially expressed in at least one mutant) and by SMa0760 and SMa0766 (100% of the genes differentially expressed in at least one mutant). Intriguingly, the transcriptional response in this region was exclusive to the PHB cycle mutants and did not include the *glgA1* mutant, which implies that the response is specific to mutation in the PHB cycle.

10.1128/mSystems.00035-17.3 FIG S3  Between-strain differential expression (log_2_-fold change) of each gene in *S. meliloti* arranged in order of ORF position in each genetic element under nitrogen-limited conditions. Download FIG S3, PDF file, 1.4 MB. Copyright © 2017 D’Alessio et al. 2017 D’Alessio et al. This content is distributed under the terms of the Creative Commons Attribution 4.0 International license.

10.1128/mSystems.00035-17.4 FIG S4  Between-strain differential expression (log_2_-fold change) of each gene in *S. meliloti* arranged in order of ORF position in each genetic element under balanced conditions. Download FIG S4, PDF file, 1.4 MB. Copyright © 2017 D’Alessio et al. 2017 D’Alessio et al. This content is distributed under the terms of the Creative Commons Attribution 4.0 International license.

To connect the discovery of these loci to our initial analysis in the Rm1021 background, we took a closer look at the differential expression in Rm1021 under balanced and nitrogen-limited conditions. We focused on the 120-kb region, as it has the largest number of genes, at 124. Of the genes in the 120-kb region, the expression of 52 differed under the two conditions. Most of these genes were upregulated in the wild type under nitrogen-limited conditions, and many of them were strongly differentially expressed in the mutants compared to the wild type (Fig. S5). The genes upregulated in the wild type under nitrogen-limited conditions tend to be downregulated in the mutants compared to the parental strain under the same conditions. We hypothesize that this may be due to an inability or impairment of the mutants to respond to nitrogen limitation. This would be an interesting line of inquiry for future work.

10.1128/mSystems.00035-17.5 FIG S5  Log_2_-fold changes in between-strain differential expression in the 120-kb region under both conditions alongside the within-strain differential expression levels. Download FIG S5, EPS file, 1.8 MB. Copyright © 2017 D’Alessio et al. 2017 D’Alessio et al. This content is distributed under the terms of the Creative Commons Attribution 4.0 International license.

The 120-kb region contains a smaller number of genes that were downregulated than of genes that were upregulated in Rm1021 under nitrogen-limited conditions. The nine downregulated genes were SMa1099, SMa1182, SMa1183, SMa1184, SMa1213, SMa1214, SMa1269, SMa1272, and SMa1273. These genes encode a hypothetical protein with sequence similarity to the cytochrome *c* oxidase genes, the *nos* operon (although the *nosR* regulator was upregulated), *fixQ1/fixP1*, and the *nor* operon. These downregulated genes may reflect the metabolism of the cell under nitrogen-limited conditions, where denitrification would be unlikely to be supported. In contrast to upregulated genes, the strongly downregulated genes tend to show similar responses in the parental and mutant backgrounds (Fig. S5).

The 120-kb region is rich in genes that encode proteins involved in nitrogen utilization, symbiotic nitrogen fixation, and denitrification. This includes the FixLJ two-component regulatory system that regulates N_2_ fixation in symbiosis in response to oxygen levels, through its adjacent Fix cluster, including the FixK1 transcriptional regulator. Also included are the NirK nitrite reductase, Nap periplasmic nitrate reductase, Nos nitrous oxide reductase, and Nor nitric oxide reductase operons, all of which have been implicated in denitrification (63). No genes in this region have previously been associated with PHB metabolism or with a regulator known to act on targets other than nitrogen utilization genes.

The 37-kb region from SMa0631 to SMa0697 is located downstream of the *fixI2S2* operon and contains mostly conserved/hypothetical genes, including SMa0662, which is a putative Fnr/Crp regulator. This region also includes the *arc* operon, which encodes arginine transport and metabolism proteins, consistent with the effects of nitrogen levels on PHB regulation. Also in this region is SMa0669, which is predicted to encode an HlyD family protein that contains a biotin binding domain. Biotin has previously been associated with PHB accumulation (64).

The smallest pSymA region exhibiting differential regulation was a single seven-gene operon, SMa0760 to SMa0766, which spans the region from *fixT2* to *fixP2*. This operon is in a region that includes *nod* genes, which are involved in the plant-microbe signaling that precedes symbiosis. The *fix* genes are involved in nitrogen fixation and specifically encode parts of the cytochrome *c* oxidase protein. *fixNOQP1* mutants exhibit reduced nitrogen fixation, but redundancy of the *fixNOQP* operon prevents the abrogation of nitrogen fixation (65). The involvement of *fixT2* here is also of interest. FixT is an antikinase protein that can affect the transcription of its regulator, FixK, by affecting the phosphorylation of FixL (66).

All three regions contain numerous genes related to nitrogen metabolism. A consistent pattern among all of these regions in the mutants was lower expression in the mutant than in the wild type under nitrogen-limited conditions, while many of these genes were upregulated compared to the level in the parental strain under balanced conditions. On the basis of the strong transcriptional response in nitrogen-associated genes, we hypothesize that obstruction of the PHB cycle disrupts the ability of the cell to respond normally to nitrogen limitation, indicating that carbon storage is involved in the regulatory response to nitrogen limitation.

There are previously discovered and described regulators in these regions. It has been established that the FixL/FixJ two-component regulatory system regulates *fixK1* transcriptionally in *S. meliloti* and is central to a signal cascade leading to nitrogen fixation (67). Further work has indicated that Hfq is involved in the regulation of *fixLJ* transcription (68). FixK1 is known to downregulate its own transcription through *fixT1* activation (69). The transcription of *fixK1* itself was downregulated in the mutants under nitrogen-limited conditions, suggesting that there is inhibition of *fixK1*, which then prevents it from initiating the regulatory cascade in the region. The second copy, *fixK2*, is found in the 7-kb region. The *fixK2* gene in *Bradyrhizobium japonicum* has been studied more extensively, but little is known about the role of *fixK2* in *S. meliloti*. NnrR, which has been shown to act as a transcriptional activator of other genes in the region involved in the cellular response to NO, is also present in the region (30).

The localized effect of gene expression changes in each of these regions is common among the PHB mutant strains that we tested but, importantly, is not shared by the glycogen synthase mutant, indicating that the effect appears to be specific to disruption of the PHB carbon storage pathway rather than disruption of carbon storage in general. Some of the mutants do not produce PHB, while others accumulate significant amounts of PHB. The shared effect illuminates the difference between the function of intracellular PHB and the presence of PHB in the cell. If the cell were responding to the presence of the PHB polymer only, the effect would differ substantially between synthesis and degradation mutants. Instead, what was observed here is that disruption on either side of the PHB cycle resulted in a similar effect on transcription in the pSymA regions indicated. This suggests that the effect is related to a fully functional PHB cycle rather than the physical presence of a PHB granule.

### A discovered motif is related to the transcriptional response in pSymA. (i) Identification of putative motifs associated with differential expression.

Hierarchical-cluster analysis of the expression in the mutants produced two major clusters of genes, with one cluster further subdivided into five more clusters (Fig. 4A). The genes in each cluster represent those that show transcriptional patterns that are common to the mutants, possibly because of shared regulation. To identify potential regulatory sequences associated with the observed differential expression, *de novo* motif discovery was performed. Specifically, regions 200 bp upstream of each gene within each cluster were analyzed with MEME. All of the motifs discovered by MEME with E values below 1 × 10^−15^ are reported (Fig. S6).

10.1128/mSystems.00035-17.6 FIG S6  Motifs resulting from *de novo* motif discovery of the gene clusters (Fig. 4). Motifs were identified with MEME. Superscript letters: a, clusters shown in Fig. 4; b, the number of sites refers to the number of genes from the cluster with a discovered motif site in its upstream region. Download FIG S6, PDF file, 0.5 MB. Copyright © 2017 D’Alessio et al. 2017 D’Alessio et al. This content is distributed under the terms of the Creative Commons Attribution 4.0 International license.

Several potential regulatory motifs were identified upstream of the genes in each expression cluster (Fig. S6). Cluster A showed the strongest transcriptional response in all of the mutants under nitrogen-limited conditions (Fig. 4B). The most significant (E value of 3.3 × 10^−80^) and biologically intriguing motif in this cluster is shown in Fig. 4C. This motif was compared to a library of known motifs by using Tomtom, which identified a close match to the previously identified Fnr/Crp family FixK transcription factor binding motif (Fig. 4C). We therefore will refer to the cluster A motif as the discovered motif. The first transcription factor and motif of this family was discovered in *Escherichia coli* and termed Fnr (fumarate and nitrate reductase). The Fnr regulatory system plays a role in anaerobic regulatory responses (67, 70, 71). More recent work has found Fnr family genes in *Rhizobiales* and termed this subgroup of proteins FixK. FixK proteins are a subset of the Fnr/Crp regulatory family, and their identified motifs, while varied, generally show the same pattern as Fnr proteins. Interestingly, multiples of these homologs have been previously identified in *S. meliloti*, as FixK1, FixK2, and NnrR, and all are involved in the regulation of nitrogen fixation during symbiosis and the response to NO (67, 72). The FixK consensus sequence has been identified in previous work as TTGAT-N_4_-ATCAA (69). NnrR also has a consensus binding sequence, identified as TTG-N8-CAAA, that has been previously hypothesized to share homology with FixK and may, in fact, overlap the FixK consensus sequence (30). Both FixK1 and NnrR are homologs of the Fnr transcriptional activator in *E. coli* (72). This suggests that a system closely resembling the Fnr regulatory system in *E. coli* may be responsible for the observed transcriptional response in the PHB cycle mutants under nitrogen-limited conditions.

**FIG 5:**
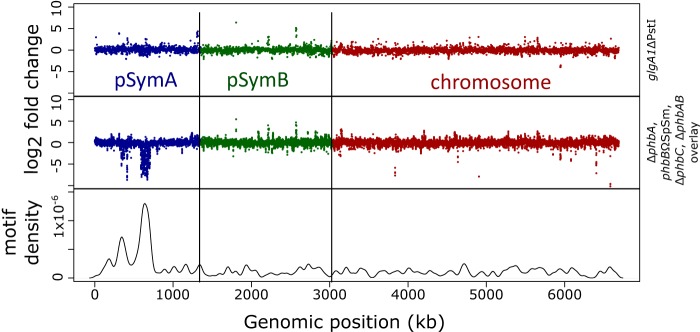
Density of the discovered motif throughout the Rm1021 genome, aligned with a composite of the PHB synthesis mutants, and between-strain differential expression of *glgA1* under nitrogen-limited conditions.

The density of the discovered motif across the entire genome was mapped after the discovered motif was identified across the genome by using the coordinates of each motif location and compared against the expression profile of the PHB synthesis mutants and the *glgA1* mutant under nitrogen-limited conditions (Fig. 5). This showed a strong correlation between the density of occurrences of the discovered motif in the genome and the transcriptional effect seen in the PHB cycle mutants under nitrogen-limited conditions. That is, the region with the highest motif density overlaps the identified 120-kb region of pSymA associated with the strong decrease in transcript abundance. This finding supports the idea that the hypothesized mechanism of Fnr-mediated regulation is responsible for the observed transcriptional response in the pSymA regions. This correlation of density and transcriptional effect does not apply to the *glgA1* mutant, suggesting that this region is regulated in response to PHB and not glycogen accumulation. In addition, it has been previously demonstrated that bacterial transcription factors tend to act locally, which suggests that the relevant Fnr/Crp regulator may be located in the region itself (73, 74).

### (ii) The discovered motif colocates with differential transcription in the PHB cycle mutants under nitrogen-limited conditions.

**FIG 6:**
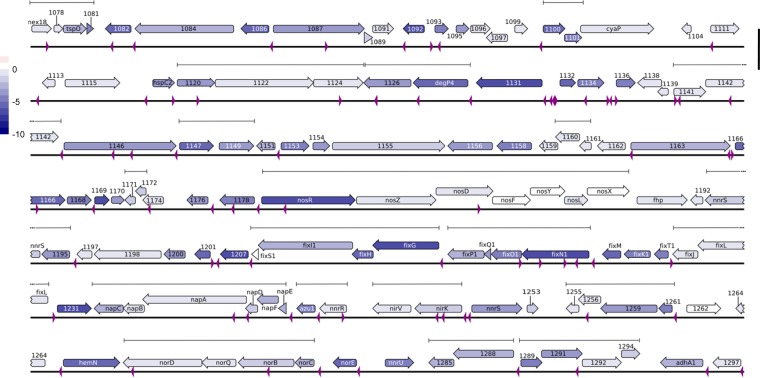
Genomic map of the 120-kb region with the discovered motif annotated, the log_2_-fold change average of the PHB synthesis mutants mapped to each gene, and transcriptional operons denoted. The bar represents a length of 1 kb.

**FIG 7:**
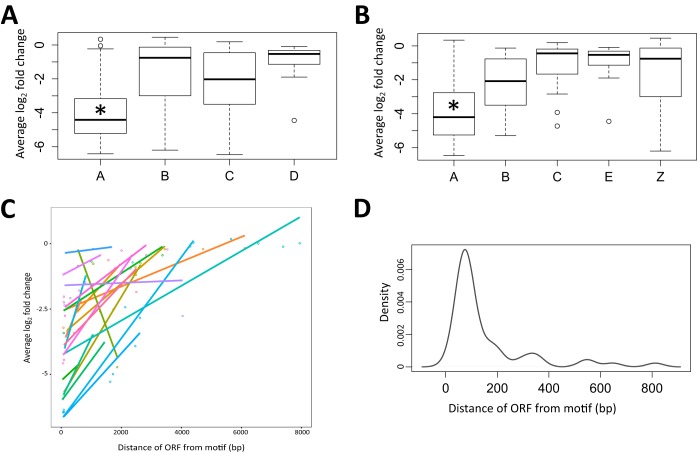
Presence of motifs correlated with decreased expression of downstream genes. (A) Single transcription units with a predicted motif upstream (A on the *x* axis) show a decreased average log_2_-fold change in the PHB synthesis mutants under nitrogen-limited conditions compared to transcripts with no predicted motif upstream (B on the *x* axis), operons with a motif upstream (C on the *x* axis), and operons without a motif upstream (D on the *x* axis). (B) Transcripts with a predicted motif upstream that are first-order genes (A on the *x* axis) show a decreased average log_2_-fold change in the PHB synthesis mutants compared to transcripts from a second-order gene (B on the *x* axis) or a third-order gene (C on the *x* axis) with a motif upstream and operons (E on the *x* axis) and single transcripts (Z on the *x* axis) with no motif upstream. Asterisks denote statistically significant differences (*P* > 0.001). (C) Effect size (average log_2_-fold change) for transcripts in PHB synthesis mutants shows signal decay in operons with a predicted motif upstream (each dot represents a gene, and colored connected dots denote genes in the same operon). (D) First-order genes with a predicted motif upstream tend to be <200 bp away from the predicted motif.

The overrepresentation of the discovered motif in regions with a strong transcriptional effect is compelling evidence of a putative regulatory system that is influenced by the PHB cycle. To further investigate this, we identified all of the instances of the predicted motif across the 120-kb region and examined the log_2_-fold changes in the expression of the associated downstream genes and operons (Fig. 6). The transcriptional effects in the three regions were strongest and most consistent between the PHB synthesis (*phbA*, *phbB*, *phbC*, and *phbAB*) mutants under nitrogen-limited conditions. It can be qualitatively observed that the genes showing the strongest effect size occurred directly downstream of a predicted motif. We then manually curated genes into four groups (A, genes with motif upstream; B, genes without a motif upstream; C, operons with a motif upstream; D, operons without a motif upstream) to perform a significance analysis of the effect of a motif on the expression of the downstream gene. The average log_2_-fold change in genes with a motif upstream was found to be significantly lower than that in all of the other groups (Fig. 7A). In addition, we observed a striking trend in operons with a motif upstream where the first gene was strongly downregulated but each gene further down the operon had a decreased effect size. To study this effect further, we then classified genes into five groups (A, first-order genes with a motif upstream; B, second-order genes with a motif upstream of the operon; C, third-order genes with a motif upstream of the operon; E, operons with no motif upstream; Z, first-order genes without a motif upstream) for further significance analysis. The average log_2_-fold change in first-order genes with a motif upstream was found to be lower than that of all of the other groups (Fig. 7B). A further reduction of the effect size was seen among groups A, B, and C. This trend was observed only in operons in the three regions that had a motif upstream and not in operons with no motif (Fig. 7C). This interesting effect of gene expression intensity across operons is a known phenomenon and has been discussed to some degree in the literature (75). To determine the common location of the motif, the distance from the open reading frame (ORF) to the closest upstream motif was mapped (Fig. 7D). The motif was found to occur most often within the first 50 bp upstream of an ORF, consistent with a transcriptional regulatory function. When the locations of the motifs are compared to recently published TSS data on *S. meliloti*, there is close alignment of many of the motifs with mRNA TSS (mTSS) positions (Fig. S1D) (33). These analyses strongly support the hypothesis that the discovered motif represents the sites for transcriptional regulator binding that influence the expression of genes affected in the PHB cycle mutants.

### (iii) Enrichment of Fnr homologs in the pSymA regions.

Recognizing that the Fnr-like motif is enriched in the pSymA regions of interest, we searched for potential Fnr-like regulators encoded in these regions. A Pfam search of the *S. meliloti* Rm1021 proteome for the main Fnr family domain (HTH_Crp2 [PF13545]) revealed 10 predicted-gene-encoded proteins with this domain. The HTH_Crp2 domain is an annotation of a helix-turn-helix pattern found in the Crp protein of *E. coli*. Further searching by using the cNMP_binding (PF00027) domain identified another four predicted proteins with the cNMP_binding domain, including an HTH_Crp domain that had escaped annotation in the Pfam database. In total, there were 14 genes predicted to encode proteins in the Crp/Fnr family in *S. meliloti* Rm1021 (Table S2). Of these, four are in the 120-kb region of pSymA, which is a 15.9-fold overrepresentation over that expected on the basis of genome-wide occurrence. Additional Fnr-related family members reside in each of the two smaller regions, i.e., *fixK2* in the 7-kb region and SMa0662 in the 37-kb region.

10.1128/mSystems.00035-17.10 TABLE S2  Fnr/Crp-encoding genes in *S. meliloti* Rm1021 identified through the presence of two key domains. Download TABLE S2, DOCX file, 0.01 MB. Copyright © 2017 D’Alessio et al. 2017 D’Alessio et al. This content is distributed under the terms of the Creative Commons Attribution 4.0 International license.

As discussed earlier, the 120-kb region includes the previously identified FixK1 and NnrR proteins but also contains SMa1141 and SMa1207, which are both putative Fnr/Crp regulatory proteins. Of these, FixK1 is a known regulator of nitrogen fixation in *S. meliloti* and NnrR is a regulator of the *nap*, *nir*, and *nor* operons (72, 76). The *fixK2* gene in the 7-kb region encodes another characterized Fnr regulator, but its specific role in the regulation of the *fix* operons in *S. meliloti* is not known. There is no information available regarding a regulatory role for SMa0662 in the 37-kb region.

While all six proteins were predicted to be structurally related to Fnr, they have one key difference in common. Unlike the originally identified Fnr protein in *E. coli*, they do not possess an N-terminal cysteine-rich region. This region allows Fnr to respond to oxygen (also NO) levels in the cell through an oxygen-labile Fe-S cluster in the N-terminal region. This cluster undergoes conversion upon oxygen binding, and the protein is unable to bind to DNA and act as a transcriptional activator (77). Since the six Fnr homologs in the pSymA regions are expected to be unable to detect cellular levels of oxygen, their function must be dependent on reaction to the presence of another compound or environmental condition or activation by another protein or be transcriptionally regulated. It has been previously established that *fixK1* transcription is regulated by FixLJ (in the presence/absence of oxygen) and by FixT1 (69, 78). NnrR induces a regulatory response in its associated genes in response to NO, but no mechanism has been elucidated (72).

Comparison and sequence alignment of the six Fnr/Crp proteins identified showed that five (SMa1207 excluded) contain a conserved, cyclic mononucleotide (cNMP) binding domain (Fig. S7). This is a trait shared with Crp, whose regulatory activity in *E. coli* is well known to be controlled by the binding of cyclic AMP (cAMP) to induce a catabolite repression response (79). The cNMP binding domain in the Fnr/Crp proteins suggests a regulatory role for cNMPs in the regulation of the pSymA regions through the involvement of cAMP or another cNMP, although this has not been studied in Fnr/Crp proteins in *Rhizobiales*. This would involve intracellular signaling to modulate the activity of these regulators as a reflection of the overall metabolic state of the cell. No experimental work has been published in support of this hypothesis. There is a known instance of catabolite repression in *S. meliloti* which is modulated through the incomplete phosphotransferase system to favor the use of succinate over many other carbon sources (80). Succinate-mediated catabolite repression is regulated separately from cAMP levels, but this does not discount the possibility of other regulatory systems with cAMP or other cyclic mononucleotides (81). Previous work with *S. meliloti* Rm2011 showed a decrease in the abundance of *fixK1* when cAMP was overproduced by overexpressed *cyaJ*, indicating a plausible relationship between internal cAMP levels and *fixK1* expression (32). No other genes in the three pSymA regions were affected in the *cyaJ* overexpression study, so the cAMP concentration in the wild type is not enough to trigger the transcriptional effect observed in these regions (SMa1082 excepted). Overall cAMP levels are reflective of the cell’s metabolism and thus would be useful in modulating a regulatory system that responds to internal levels of carbon and/or nitrogen. A speculative model is that disruption of the PHB cycle interferes with cellular respiration and central metabolism, which may lead to alteration of the intracellular levels of cAMP, which could act as a ligand for an Fnr protein that regulates genes in the three pSymA regions identified.

10.1128/mSystems.00035-17.7 FIG S7  Alignment of the six Fnr/Crp proteins located in the pSymA loci showing shared protein domains. Download FIG S7, EPS file, 0.5 MB. Copyright © 2017 D’Alessio et al. 2017 D’Alessio et al. This content is distributed under the terms of the Creative Commons Attribution 4.0 International license.

### Conclusions.

We applied transcriptomic profiling to PHB cycle and glycogen pathway disruption mutants to identify and amplify the signal associated with the synthesis and degradation of PHB, as distinguished from individual gene mutation effects. Combined with computational genomic analysis, this revealed three distinct regions of pSymA that contain genes whose expression under nitrogen-limited conditions is impacted by PHB cycle disruption but not glycogen pathway disruption. These regions of pSymA contain genes involved in nitrogen uptake, nitrogen utilization, symbiotic nitrogen fixation, and denitrification, underlining the interrelation of PHB accumulation and nitrogen levels in the cell. We hypothesize that PHB cycle disruption results in an inability of the cell to respond appropriately to nitrogen limitation. Further examination of the genes whose expression was impacted in PHB cycle mutants led to the discovery of an associated motif that closely resembles the previously discovered FixK motif. The density of this motif was shown to correlate specifically with the genome regions with the greatest transcriptional change, and the presence of the motif upstream of genes in these regions correlates with the decreased gene expression level in the PHB synthesis mutants under nitrogen-limited conditions. These regions encode a high density of candidate Fnr/Crp family regulators that likely bind similar motifs. In the absence of any published findings that can explain the transcriptional effects seen, we speculate that intracellular cAMP levels in the PHB cycle mutants may be altered, leading to the modification of an Fnr/Crp family protein, which results in the downregulation of genes in three regions of pSymA through transcription factor binding of the motif discovered.

New questions that arise from this work include the nature of the mechanism of gene regulation in the regions identified; how gene regulation is influenced by PHB synthesis; whether intracellular cAMP levels are affected in the PHB cycle mutants; whether the local enrichment of proteins carrying the motif discovered is involved in fine-tuning of the regulatory response; and how this regulatory effect is connected to sensing of the carbon/nitrogen ratio. We hypothesize that one or more of the Fnr/Crp family proteins are required for some of the differential expression that is observed, and this can be tested through subsequent experiments.

## MATERIALS AND METHODS

### Bacterial strains, media, antibiotics, recombinant DNA, and bacterial genetics.

The strains and plasmids used in this study are listed in Table 1. Bacteria were cultured under previously described conditions, in TY or LB with appropriate antibiotics (82). The antibiotics used with *S. meliloti* were neomycin (200 µg/ml) and streptomycin (200 µg/ml); those used with *E. coli* were kanamycin (25 µg/ml) and ampicillin (100 µg/ml). The deletion mutations of *phbAB*, *phbC*, and *phaZ* were carried out in a similar manner, by utilizing pK19*mobsacB* selection of homologous recombination on sucrose. For *phbAB*, *phbC*, and *phaZ*, 400-bp synthetic fragments that contained the 200 bp immediately up- and downstream of the corresponding ORF were used. These fragments were synthesized by BioBasic and delivered in purified pUC57. The inserts were subcloned from their vectors into pK19*mobsacB* as EcoRI/XbaI fragments, and the resulting constructs were transferred to Rm1021 by triparental conjugation. Neomycin selection resulted in integration of the plasmid into the chromosome via single crossover. Selection with 5% sucrose selected for resolution, resulting in revertants or double-crossover deletion mutants. The colonies were screened for neomycin sensitivity to confirm the loss of the plasmid. Confirmed clones were screened by colony PCR to differentiate between revertants and deletion mutants. The resulting strains were SmUW234 (Δ*phbAB*), SmUW235 (Δ*phbC*), and SmUW236 (Δ*phaZ*).

**TABLE 1:** Bacterial strains, plasmids, and primers used in this study

Strain, plasmid, or primer	Relevant characteristic(s) or sequence	Reference
*S. meliloti*		
Rm1021	SU47 *str-21* Sm^r^, wild type	91
Rm11134	Rm1021 *acsA2*::Tn*5* Sm^r^ Nm^r^	92
Rm11347	Rm1021 *phbB*::ΩSpSm Sm^r^ Sp^r^	93
Rm11401	Rm1021 Δ*bdhA* (in-frame deletion 1-3) Sm^r^	58
SmUW41	Rm1021 Δ*phbA* Sm^r^	This work
SmUW234	Rm1021 Δ*phbAB* Sm^r^	This work
SmUW235	Rm1021 Δ*phbC* Sm^r^	This work
SmUW236	Rm1021 Δ*phaZ* Sm^r^	This work
Rm11479	Rm1021 *glgA1*Δ PstI Sm^r^	15
		
*E. coli*		
DH5α	F^−^ *endA1 glnv44 thi-1 recA1 relA1 gyrA96 deoR* *nupG purB20* φ80d*lacZ*ΔM15 Δ(*lacZYA-argF*)*U169* *hsdR17*(r_K_^−^ m_K_^+^) λ^−^	94
MT616	MT607/pRK600, mobilizer	95
		
Plasmids		
pK19*mobsacB*	Suicide vector, *sacB*^+^, *mobRK2*, *ori*_R6K_, Nm-Km^r^	96
pRK600	Mobilizer plasmid for conjugal transfer, Cm^r^ Nm-Km^s^	95
pUC57	Harbors synthesized constructs	
pMA188	pGEMTEasy carrying *phbA* deletion crossover PCR product	62
pMA190	pK19*mobsacB* carrying *phbA* deletion crossover PCR product subcloned from pMA188	62
pGEMTEasy		Promega
		
Primers		
SMc03879A	CGCGAATTCATCAACTGGCGGAGAAAG	
SMc03879B	TGCGAGGATCAGGTCGTGGTGGGGACGTTCCTCATATTTG	
SMc03879C	CACCACGACCTGATCCTCGCAGAGTGGGAGGCGAGTATG	
SMc03879D	GCTCTAGAGCCGGAAAGCCGAGGGCAC	
SMc03879F	CGCAAGCTTCATGAGCAATCCCTCGATC	
SMc03879R	GCTCTAGATTACAGGCGTTCCACGCAC	

The RNA-seq analysis reported here confirmed that no transcript (deletion mutant) or no complete transcript (insertion mutant) corresponding to the deleted gene(s) of each of the mutants was detectable.

In preparation for RNA extraction, 5-ml cultures were grown to saturation in tryptone-yeast extract broth. The cells were washed twice and resuspended in an equivalent volume of 0.85% (wt/vol) NaCl. Aliquots (750 μl) of washed cells were used to inoculate 250-ml baffled shake flask cultures containing 75 ml of broth. Nitrogen-limited medium consisted of 5 g of mannitol, 0.5 g of yeast extract, 1 g of K_2_HPO_4_, 0.2 g of MgSO_4_ ⋅ 7H_2_O, 0.1 g of NaCl, 0.5 g of CaCl_2_ ⋅ 2H_2_O, and 0.004 g of FeCl_3_ ⋅ 6H_2_O/liter, adjusted to pH 7.2. Balanced medium was the same but had 20 times as much yeast extract (10 g).

### RNA extraction.

Antibiotics were not used in cultures from which RNA was extracted, to avoid transcriptomic bias introduced by antibiotic presence. Cultures were shaken at 200 rpm at 30°C. Cells were harvested at mid- to late-log phase as indicated by optical density at 600 nm readings of 0.5 ± 0.05 (Fig. S1). To harvest cells, 45 ml of culture broth was transferred to a sterile container and 5 ml of stop solution (5% phenol in absolute ethanol) was added. The cells were collected by centrifugation at 6,000 × *g* and 4°C for 10 min, and the liquid was decanted. Pellets were frozen in liquid nitrogen and stored at −80°C for up to 2 months prior to RNA isolation.

RNA was extracted by the hot phenol method, much as previously described (83). Briefly, cells were lysed and total nucleic acids were initially quantified with a NanoDrop. Equivalent amounts of total nucleic acids were then compared with DNA standards of known quantity to determine the amount of contaminating genomic DNA. The samples were then treated with 1 U of RNase-free DNase I/µg of DNA and 40 U of RiboLock/µg of RNA. The RNA was cleaned up with the RNeasy minikit (Qiagen), and RNA quality was determined by electrophoresis on an agarose-formaldehyde denaturing gel stained with ethidium bromide. The RNA concentration was determined with a NanoDrop 1000 (Thermo Fisher), and subsamples from isolates with >1 µg/µl were run on agarose gels. If the 16S rRNA band was approximately half as bright as the 23S rRNA band, the samples were used for RNA-seq analysis.

### RNA preparation for sequencing.

rRNA depletion was performed with 1 µg of total RNA and the Meta-Bacteria Ribo-Zero rRNA Removal kit (Mandel Scientific, Guelph, Ontario, Canada) at the Genome Quebec Innovation Center of McGill University. Final RNA was isolated with the RiboMinus Concentration Module and eluted with 17 µl of Fragment, Prime, Finish buffer (Life Technologies, Inc.). The eluted RNA was then fragmented and prepared for cDNA synthesis. The TruSeq Stranded mRNA Sample Prep kit was used for cDNA synthesis, and then the quality of the library and fragment size were assessed with a LabChip, a LightCycler 480 II, and an Infinite M200 Fluorimeter (Roche, Mississauga, Ontario, Canada, and Tecan, Mannedorf, Switzerland).

### Illumina sequencing and raw-read processing.

The cDNA was sequenced (2 × 100 bp) on an Illumina HiSeq 2000 instrument at the Genome Quebec Innovation Center of McGill University. There was an average of 21,592,337 reads with an average Phred quality score of 35 for each sample. The average number of base pairs per sample was 4,318,467,357. Trimmomatic was used to strip the barcode from the raw reads by using the default settings (84). TopHat was used to assemble reads to the reference genome; reads with more than two mismatches, indels, or gaps were not used (85). Cuffdiff was used to compare the normalized transcript count data (fragments per kilobase per million reads [FPKM] transcript normalization was used for comparison of samples) for each mutant to those for the wild type under the same growth conditions. The average FPKM values of the two samples were used to calculate the log_2_-fold change. Standard *t*-test analysis was performed with false-discovery rate correction. Significance was reported if the *P* value was greater than the false-discovery rate (*q* value) after Benjamini-Hochberg multiple-test correction (86). Ten sequences aligned with each gene was required as the minimum threshold to allow testing; otherwise, no test was performed (significance displays as “no”). Only transcripts that were significantly different in the mutant and parental strains were considered in the following analysis. For the differential-expression data obtained, see http://www.bitbucket.org/mdelow/phbrnaseq/.

### Motif discovery and comparison.

The 200 bp upstream of every gene in the clusters delineated on the heat map were extracted, along with the 200 bp upstream of every gene in the *S. meliloti* Rm1021 genome, with RSAT (87). These files were used to perform *de novo* motif discovery with MEME (88). The top resulting motifs were mapped to the pSymA regions with FIMO (89). The motif outputs from MEME under a *P* value threshold of 1 × 10^−15^ were input into Tomtom for comparison to the prokaryotic motif databases (90). The Tomtom output results under a *P* value threshold of 0.001 were considered and are reported here.

### Statistical analysis.

Analysis of variance was performed by using the average log_2_-fold values for each of the manually curated groups. This was done in R with the *stat.*aov, TukeyHSD, and HSD.test functions.

### Figure generation.

Figures 2 to 7 were generated in part through data visualization in R. The scripts and data files used in R for all data visualization are available as discussed in the paragraph on data availability below.

Figures 2 and 5 used the starting coordinate of each ORF to anchor the log_2_-fold change (*y*-axis value) along the *x* axis based on genomic location.

For Fig. 4, S2, and S8, hierarchical clustering of log_2_ expression values was performed with the hclust() function within R based on “complete linkage” clustering based on euclidean distances. To identify expression clusters, we used a semiautomated approach in which expression clusters were visually identified and fixed thresholds were selected to define cluster membership. At the highest level, the gene expression dendrogram split into two large clusters, A and B (see Fig. S8). Cluster A was identified as a biologically relevant cluster and was thoroughly investigated in this work. Expression cluster B was larger but had weaker expression patterns and subtle differences between subclusters, and we were unable to identify enriched motifs at the same level of significance as in cluster A. Therefore, it was further subdivided into subclusters with a lower threshold of 7, and these subclusters were analyzed further. Lastly, cluster 4 was also subdivided into two subclusters with noticeable differences in expression.

10.1128/mSystems.00035-17.8 FIG S8  Dendrogram used to set the threshold and delineate cluster groups in a hierarchical-cluster heat map of log_2_ expression data (Fig. 4; Fig. S2). Download FIG S8, EPS file, 0.4 MB. Copyright © 2017 D’Alessio et al. 2017 D’Alessio et al. This content is distributed under the terms of the Creative Commons Attribution 4.0 International license.

Figure 5 visualized motif density along the genome. First, the FIMO tool was used to detect all instances of the discovered motif genome-wide. The coordinates of each motif location were then used to visualize motif density along the genome by using R’s density() function with a scaling bandwidth parameter (b.w.) of 30,000.

Figure 6 was generated in Geneious by using the Rm1021 annotation to visually map the locations of genes in the 120-kb region with the locations matching the “discovered” motif overlaid. Further editing was done in Inkscape to change the color of each gene arrow to correspond to the log_2_-fold change. A basic heat map of the average log_2_-fold change in the expression of genes in the 120-kb region in the PHB synthesis mutants under nitrogen-limited conditions was produced in R, and the colors of the tiles were mapped onto the gene arrows in the Geneious output. No changes in gene or motif locations were made.

The statistical analysis used for Fig. 7A and B is described above. Genes were manually curated into groups based on the direct presence of a motif upstream of the ORF (the motif was assumed to be functional in either direction because of its palindromic nature) without any other intervening genetic elements. Genes with multiple motifs upstream were not treated any differently. Motifs that occurred within genes were ignored. For Fig. 7A, operons were treated as a single group, with the presence of a motif being tested upstream of the first gene in the operon. For Fig. 7B, the first gene in an operon was separated into another group from the downstream genes in the operon, but the classification of these groups was still dependent on the presence of a motif upstream of the first gene in the operon. For Fig. 7D, we used a kernel density plot to visualize the distance between upstream motifs and the closely associated ORF. This was done by using the R plot(density) function with a line width of 2.

Figure S1D and Fig. 6 were produced in Geneious. Figure S1D used the same reference as Fig. 6, with the TSS data layered on top (33). Figure S7 was produced by using the align utility on the protein sequences of the associated genes of interest.

### Data availability.

The raw RNA sequence data obtained in this study are available through the Sequence Read Archive (SRA) through BioProject accession number PRJNA369802 and associated SRA and BioSample accession numbers, titled “Analysis of the transcriptional response to mutations in PHB cycle genes in *Sinorhizobium meliloti* reveals disproportionately affected regions on pSymA containing nitrogen utilization genes.” The BioSample accession numbers (36) with the analysis data and R script are available at https://bitbucket.org/mdelow/phbrnaseq.

## References

[1] Mus F, Crook MB, Garcia K, Garcia Costas A, Geddes BA, Kouri ED, Paramasivan P, Ryu MH, Oldroyd GED, Poole PS, Udvardi MK, Voigt CA, Ané JM, Peters JW 2016 Symbiotic nitrogen fixation and the challenges to its extension to nonlegumes. Appl Environ Microbiol 82:3698–3710. doi:10.1128/AEM.01055-16. 27084023 PMC4907175

[2] Nelson MS, Sadowsky MJ 2015 Secretion systems and signal exchange between nitrogen-fixing rhizobia and legumes. Front Plant Sci 6:491. doi:10.3389/fpls.2015.00491. 26191069 PMC4486765

[3] Peters NK, Long SR 1988 Alfalfa root exudates and compounds which promote or inhibit induction of *Rhizobium meliloti* nodulation genes. Plant Physiol 88:396–400. doi:10.1104/pp.88.2.396. 16666315 PMC1055588

[4] Caetano-Anollés G, Wall LG, De Micheli AT, Macchi EM, Bauer WD, Favelukes G 1988 Role of motility and chemotaxis in efficiency of nodulation by *Rhizobium meliloti*. Plant Physiol 86:1228–1235. doi:10.1104/pp.86.4.1228. 16666059 PMC1054656

[5] Gage DJ 2002 Analysis of infection thread development using Gfp- and DsRed-expressing *Sinorhizobium meliloti*. J Bacteriol 184:7042–7046. doi:10.1128/JB.184.24.7042-7046.2002. 12446653 PMC135452

[6] Trainer MA, Charles TC 2006 The role of PHB metabolism in the symbiosis of rhizobia with legumes. Appl Microbiol Biotechnol 71:377–386. doi:10.1007/s00253-006-0354-1. 16703322

[7] Hirsch AM, Bang M, Ausubel FM 1983 Ultrastructural analysis of ineffective alfalfa nodules formed by *nif*::Tn*5* mutants of *Rhizobium meliloti*. J Bacteriol 155:367–380. 6575011 10.1128/jb.155.1.367-380.1983 PMC217689

[8] Aneja P, Charles TC 1999 Poly-3-hydroxybutyrate degradation in *Rhizobium* (*Sinorhizobium*) *meliloti*: isolation and characterization of a gene encoding 3-hydroxybutyrate dehydrogenase. J Bacteriol 181:849–857. 9922248 10.1128/jb.181.3.849-857.1999 PMC93451

[9] Aneja P, Zachertowska A, Charles TC 2005 Comparison of the symbiotic and competition phenotypes of *Sinorhizobium meliloti* PHB synthesis and degradation pathway mutants. Can J Microbiol 51:599–604. doi:10.1139/w05-042. 16175209

[10] Peoples OP, Sinskey AJ 1989 Fine structural analysis of the *Zoogloea ramigera* *phbA-phbB* locus encoding β-ketothiolase and acetoacetyl-CoA reductase: nucleotide sequence of *phbB*. Mol Microbiol 3:349–357. doi:10.1111/j.1365-2958.1989.tb00180.x. 2546004

[11] Peoples OP, Sinskey AJ 1989 Poly-β-hydroxybutyrate (PHB) biosynthesis in *Alcaligenes eutrophus* H16. Identification and characterization of the PHB polymerase gene (phbC). J Biol Chem 264:15298–15303. 2670936

[12] Schubert P, Steinbüchel A, Schlegel HG 1988 Cloning of the *Alcaligenes eutrophus* genes for synthesis of poly-β-hydroxybutyric acid (PHB) and synthesis of PHB in *Escherichia coli*. J Bacteriol 170:5837–5847. doi:10.1128/jb.170.12.5837-5847.1988. 2848014 PMC211690

[13] Delafield FP, Doudoroff M, Palleroni NJ, Lusty CJ, Contopoulos R 1965 Decomposition of poly-β-hydroxybutyrate by pseudomonads. J Bacteriol 90:1455–1466. 5848334 10.1128/jb.90.5.1455-1466.1965 PMC315835

[14] Cai GQ, Driscoll BT, Charles TC 2000 Requirement for the enzymes acetoacetyl coenzyme A synthetase and poly-3-hydroxybutyrate (PHB) synthase for growth of *Sinorhizobium meliloti* on PHB cycle intermediates. J Bacteriol 182:2113–2118. doi:10.1128/JB.182.8.2113-2118.2000. 10735852 PMC111258

[15] Wang C, Saldanha M, Sheng X, Shelswell KJ, Walsh KT, Sobral BWS, Charles TC 2007 Roles of poly-3-hydroxybutyrate (PHB) and glycogen in symbiosis of *Sinorhizobium meliloti* with *Medicago* sp. Microbiology 153:388–398. doi:10.1099/mic.0.29214-0. 17259610

[16] Trainer MA, Capstick D, Zachertowska A, Lam KN, Clark SRD, Charles TC 2010 Identification and characterization of the intracellular poly-3-hydroxybutyrate depolymerase enzyme PhaZ of *Sinorhizobium meliloti*. BMC Microbiol 10:92. doi:10.1186/1471-2180-10-92. 20346169 PMC2867953

[17] Wang C, Sheng X, Equi RC, Trainer MA, Charles TC, Sobral BWS 2007 Influence of the poly-3-hydroxybutyrate (PHB) granule-associated proteins (PhaP1 and PhaP2) on PHB accumulation and symbiotic nitrogen fixation in *Sinorhizobium meliloti* Rm1021. J Bacteriol 189:9050–9056. doi:10.1128/JB.01190-07. 17921298 PMC2168632

[18] Wang C, Kemp J, Da Fonseca IO, Equi RC, Sheng X, Charles TC, Sobral BWS 2010 *Sinorhizobium meliloti* 1021 loss-of-function deletion mutation in *chvI* and its phenotypic characteristics. Mol Plant Microbe Interact 23:153–160. doi:10.1094/MPMI-23-2-0153. 20064059

[19] Damrow R, Maldener I, Zilliges Y 2016 The multiple functions of common microbial carbon polymers, glycogen and PHB, during stress responses in the non-diazotrophic cyanobacterium *Synechocystis* sp. PCC 6803 Front Microbiol 7:966. doi:10.3389/fmicb.2016.00966. PMC4914499 27446007

[20] Tsien HC, Schmidt EL 1977 Polarity in the exponential-phase *Rhizobium japonicum* cell. Can J Microbiol 23:1274–1284. doi:10.1139/m77-191. 907920

[21] Ugalde JE, Parodi AJ, Ugalde RA 2003 *De novo* synthesis of bacterial glycogen: *Agrobacterium tumefaciens* glycogen synthase is involved in glucan initiation and elongation. Proc Natl Acad Sci U S A 100:10659–10663. doi:10.1073/pnas.1534787100. 12960388 PMC196860

[22] Peplinski K, Ehrenreich A, Döring C, Bömeke M, Reinecke F, Hutmacher C, Steinbüchel A 2010 Genome-wide transcriptome analyses of the ‘Knallgas’ bacterium *Ralstonia eutropha* H16 with regard to polyhydroxyalkanoate metabolism. Microbiology 156:2136–2152. doi:10.1099/mic.0.038380-0. 20395272

[23] Shimizu R, Chou K, Orita I, Suzuki Y, Nakamura S, Fukui T 2013 Detection of phase-dependent transcriptomic changes and RuBisCO-mediated CO_2_ fixation into poly(3-hydroxybutyrate) under heterotrophic condition in *Ralstonia eutropha* H16 based on RNA-seq and gene deletion analyses. BMC Microbiol 13:169. doi:10.1186/1471-2180-13-169. 23879744 PMC3734047

[24] Ampe F, Kiss E, Sabourdy F, Batut J 2003 Transcriptome analysis of *Sinorhizobium meliloti* during symbiosis. Genome Biol 4:R15. doi:10.1186/gb-2003-4-2-r15. 12620125 PMC151305

[25] Becker A, Bergès H, Krol E, Bruand C, Rüberg S, Capela D, Lauber E, Meilhoc E, Ampe F, de Bruijn FJ, Fourment J, Francez-Charlot A, Kahn D, Küster H, Liebe C, Pühler A, Weidner S, Batut J 2004 Global changes in gene expression in *Sinorhizobium meliloti* 1021 under microoxic and symbiotic conditions. Mol Plant Microbe Interact 17:292–303. doi:10.1094/MPMI.2004.17.3.292. 15000396

[26] Tian Z, Zou H, Li J, Zhang Y, Liu Y, Yu G, Zhu J, Rüberg S, Becker A, Wang Y 2006 Transcriptome analysis of *Sinorhizobium meliloti* nodule bacteria in *nifA* mutant background. Chin Sci Bull 51:2079–2086. doi:10.1007/s11434-006-2092-2.

[27] Gibson KE, Barnett MJ, Toman CJ, Long SR, Walker GC 2007 The symbiosis regulator CbrA modulates a complex regulatory network affecting the flagellar apparatus and cell envelope proteins. J Bacteriol 189:3591–3602. doi:10.1128/JB.01834-06. 17237174 PMC1855900

[28] Chen EJ, Fisher RF, Perovich VM, Sabio EA, Long SR 2009 Identification of direct transcriptional target genes of ExoS/ChvI two-component signaling in *Sinorhizobium meliloti*. J Bacteriol 191:6833–6842. doi:10.1128/JB.00734-09. 19749054 PMC2772461

[29] Hellweg C, Pühler A, Weidner S 2009 The time course of the transcriptomic response of *Sinorhizobium meliloti* 1021 following a shift to acidic pH. BMC Microbiol 9:37. doi:10.1186/1471-2180-9-37. 19216801 PMC2651895

[30] Meilhoc E, Cam Y, Skapski A, Bruand C 2010 The response to nitric oxide of the nitrogen-fixing symbiont *Sinorhizobium meliloti*. Mol Plant Microbe Interact 23:748–759. doi:10.1094/MPMI-23-6-0748. 20459314

[31] Yurgel SN, Rice J, Kahn ML 2013 Transcriptome analysis of the role of GlnD/GlnBK in nitrogen stress adaptation by *Sinorhizobium meliloti* Rm1021. PLoS One 8:e58028. doi:10.1371/journal.pone.0058028. 23516427 PMC3596328

[32] Krol E, Klaner C, Gnau P, Kaever V, Essen LO, Becker A 2016 Cyclic mononucleotide- and Clr-dependent gene regulation in *Sinorhizobium meliloti*. Microbiology 162:1840–1856. doi:10.1099/mic.0.000356. 27535558

[33] Schlüter JP, Reinkensmeier J, Barnett MJ, Lang C, Krol E, Giegerich R, Long SR, Becker A 2013 Global mapping of transcription start sites and promoter motifs in the symbiotic α-proteobacterium *Sinorhizobium meliloti* 1021. BMC Genomics 14:156. doi:10.1186/1471-2164-14-156. 23497287 PMC3616915

[34] Torres-Quesada O, Reinkensmeier J, Schlüter JP, Robledo M, Peregrina A, Giegerich R, Toro N, Becker A, Jiménez-Zurdo JI 2014 Genome-wide profiling of Hfq-binding RNAs uncovers extensive post-transcriptional rewiring of major stress response and symbiotic regulons in *Sinorhizobium meliloti*. RNA Biol 11:563–579. doi:10.4161/rna.28239. 24786641 PMC4152363

[35] Povolo S, Casella S 2000 A critical role for *aniA* in energy-carbon flux and symbiotic nitrogen fixation in *Sinorhizobium meliloti*. Arch Microbiol 174:42–49. doi:10.1007/s002030000171. 10985741

[36] Maehara A, Doi Y, Nishiyama T, Takagi Y, Ueda S, Nakano H, Yamane T 2001 PhaR, a protein of unknown function conserved among short-chain-length polyhydroxyalkanoic acids producing bacteria, is a DNA-binding protein and represses Paracoccus denitrificans phaP expression *in vitro*. FEMS Microbiol Lett 200:9–15. doi:10.1111/j.1574-6968.2001.tb10685.x. 11410342

[37] Quelas JI, Mesa S, Mongiardini EJ, Jendrossek D, Lodeiro AR 2016 Regulation of polyhydroxybutyrate synthesis in the soil bacterium *Bradyrhizobium* *diazoefficiens*. Appl Environ Microbiol 82:4299–4308. doi:10.1128/AEM.00757-16. 27208130 PMC4942959

[38] Korotkova N, Chistoserdova L, Lidstrom ME 2002 Poly-β-hydroxybutyrate biosynthesis in the facultative methylotroph *Methylobacterium extorquens* AM1: identification and mutation of *gap11*, *gap20*, and *phaR*. J Bacteriol 184:6174–6181. doi:10.1128/JB.184.22.6174-6181.2002. 12399487 PMC151960

[39] Boncompagni E, Dupont L, Mignot T, Østeras M, Lambert A, Poggi MC, Le Rudulier D 2000 Characterization of a *Sinorhizobium meliloti* ATP-binding cassette histidine transporter also involved in betaine and proline uptake. J Bacteriol 182:3717–3725. doi:10.1128/JB.182.13.3717-3725.2000. 10850986 PMC94542

[40] Lodwig EM, Hosie AHF, Bourdès A, Findlay K, Allaway D, Karunakaran R, Downie JA, Poole PS 2003 Amino-acid cycling drives nitrogen fixation in the legume-*Rhizobium* symbiosis. Nature 422:722–726. doi:10.1038/nature01527. 12700763

[41] Yurgel SN, Rice J, Mulder M, Kahn ML 2010 GlnB/GlnK PII proteins and regulation of the *Sinorhizobium meliloti* Rm1021 nitrogen stress response and symbiotic function. J Bacteriol 192:2473–2481. doi:10.1128/JB.01657-09. 20304991 PMC2863565

[42] Sun J, Peng X, Van Impe J, Vanderleyden J 2000 The *ntrB* and *ntrC* genes are involved in the regulation of poly-3-hydroxybutyrate biosynthesis by ammonia in *Azospirillum brasilense* Sp7. Appl Environ Microbiol 66:113–117. doi:10.1128/AEM.66.1.113-117.2000. 10618211 PMC91793

[43] Liu CM, McLean PA, Sookdeo CC, Cannon FC 1991 Degradation of the herbicide glyphosate by members of the family *Rhizobiaceae*. Appl Environ Microbiol 57:1799–1804. 16348512 10.1128/aem.57.6.1799-1804.1991 PMC183471

[44] Shatters RG, Liu Y, Kahn ML 1993 Isolation and characterization of a novel glutamine synthetase from *Rhizobium meliloti*. J Biol Chem 268:469–475. 8093245

[45] Dylan T, Nagpal P, Helinski DR, Ditta GS 1990 Symbiotic pseudorevertants of *Rhizobium meliloti* *ndv* mutants. J Bacteriol 172:1409–1417. doi:10.1128/jb.172.3.1409-1417.1990. 2307652 PMC208613

[46] Wang P, Ingram-Smith C, Hadley JA, Miller KJ 1999 Cloning, sequencing, and characterization of the *cgmB* gene of *Sinorhizobium meliloti* involved in cyclic β-glucan biosynthesis. J Bacteriol 181:4576–4583. 10419956 10.1128/jb.181.15.4576-4583.1999 PMC103589

[47] Mendrygal KE, González JE 2000 Environmental regulation of exopolysaccharide production in *Sinorhizobium meliloti*. J Bacteriol 182:599–606. doi:10.1128/JB.182.3.599-606.2000. 10633091 PMC94320

[48] Glazebrook J, Walker GC 1989 A novel exopolysaccharide can function in place of the calcofluor-binding exopolysaccharide in nodulation of alfalfa by *Rhizobium meliloti*. Cell 56:661–672. doi:10.1016/0092-8674(89)90588-6. 2537152

[49] Hoang HH, Becker A, González JE, Gonza JE 2004 The LuxR homolog ExpR, in combination with the Sin quorum sensing system, plays a central role in *Sinorhizobium meliloti* gene expression. J Bacteriol 186:5460–5472. doi:10.1128/JB.186.16.5460-5472.2004. 15292148 PMC490858

[50] Marketon MM, Gronquist MR, Eberhard A, González JE 2002 Characterization of the *Sinorhizobium meliloti* *sinR/sinI* locus and the production of novel N-acyl homoserine lactones. J Bacteriol 184:5686–5695. doi:10.1128/JB.184.20.5686-5695.2002. 12270827 PMC139616

[51] Beck S, Marlow VL, Woodall K, Doerrler WT, James EK, Ferguson GP 2008 The *Sinorhizobium meliloti* MsbA2 protein is essential for the legume symbiosis. Microbiology 154:1258–1270. doi:10.1099/mic.0.2007/014894-0. 18375818

[52] Wells DH, Chen EJ, Fisher RF, Long SR 2007 ExoR is genetically coupled to the ExoS-ChvI two-component system and located in the periplasm of *Sinorhizobium meliloti*. Mol Microbiol 64:647–664. doi:10.1111/j.1365-2958.2007.05680.x. 17462014

[53] Yuan ZC, Zaheer R, Finan TM 2006 Regulation and properties of PstSCAB, a transport system of *Sinorhizobium meliloti*. J Bacteriol 188:1089–1102. doi:10.1128/JB.188.3.1089-1102.2006. 16428413 PMC1347321

[54] Persmark M, Pittman P, Buyer JS, Schwyn B, Gill PR, Neilands JB 1993 Isolation and structure of rhizobactin 1021, a siderophore from the alfalfa symbiont *Rhizobium meliloti* 1021. J Am Chem Soc 115:3950–3956. doi:10.1021/ja00063a014.

[55] Lynch D, O’Brien J, Welch T, Clarke P, Cuív PO, Crosa JH, O’Connell M 2001 Genetic organization of the region encoding regulation, biosynthesis, and transport of rhizobactin 1021, a siderophore produced by *Sinorhizobium meliloti*. J Bacteriol 183:2576–2585. doi:10.1128/JB.183.8.2576-2585.2001. 11274118 PMC95175

[56] Rüberg S, Tian ZX, Krol E, Linke B, Meyer F, Wang Y, Pühler A, Weidner S, Becker A 2003 Construction and validation of a *Sinorhizobium meliloti* whole genome DNA microarray: genome-wide profiling of osmoadaptive gene expression. J Biotechnol 106:255–268. doi:10.1016/j.jbiotec.2003.08.005. 14651866

[57] Krol E, Becker A 2004 Global transcriptional analysis of the phosphate starvation response in *Sinorhizobium meliloti* strains 1021 and 2011. Mol Genet Genomics 272:1–17. doi:10.1007/s00438-004-1030-8. 15221452

[58] Sukdeo N, Charles TC 2003 Application of crossover-PCR-mediated deletion-insertion mutagenesis to analysis of the *bdhA-xdhA2-xdhB2* mixed-function operon of *Sinorhizobium meliloti*. Arch Microbiol 179:301–304. doi:10.1007/s00203-003-0532-9. 12632261

[59] Santos MR, Cosme AM, Becker JD, Medeiros JMC, Mata MF, Moreira LM 2010 Absence of functional TolC protein causes increased stress response gene expression in *Sinorhizobium meliloti*. BMC Microbiol 10:180. doi:10.1186/1471-2180-10-180. 20573193 PMC2912261

[60] Barnett MJ, Long SR 2015 The *Sinorhizobium meliloti* SyrM regulon: effects on global gene expression are mediated by *syrA* and *nodD3*. J Bacteriol 197:1792–1806. doi:10.1128/JB.02626-14. 25777671 PMC4402393

[61] Nogales J, Domínguez-Ferreras A, Amaya-Gómez CV, van Dillewijn P, Cuéllar V, Sanjuán J, Olivares J, Soto MJ 2010 Transcriptome profiling of a *Sinorhizobium meliloti* *fadD* mutant reveals the role of rhizobactin 1021 biosynthesis and regulation genes in the control of swarming. BMC Genomics 11:157. doi:10.1186/1471-2164-11-157. 20210991 PMC2848241

[62] Trainer MA 2009 Carbon metabolism and desiccation tolerance in the nitrogen-fixing rhizobia *Bradyrhizobium**japonicum* and *Sinorhizobium**meliloti*. Ph.D. dissertation University of Waterloo, Waterloo, Ontario, Canada.

[63] Torres MJ, Argandoña M, Vargas C, Bedmar EJ, Fischer HM, Mesa S, Delgado MJ 2014 The global response regulator RegR controls expression of denitrification genes in *Bradyrhizobium* *japonicum*. PLoS One 9:e99011. doi:10.1371/journal.pone.0099011. 24949739 PMC4064962

[64] Hofmann K, Heinz EB, Charles TC, Hoppert M, Liebl W, Streit WR 2000 *Sinorhizobium meliloti* strain 1021 *bioS* and *bdhA* gene transcriptions are both affected by biotin available in defined medium. FEMS Microbiol Lett 182:41–44. doi:10.1111/j.1574-6968.2000.tb08870.x. 10612728

[65] Torres MJ, Hidalgo-García A, Bedmar EJ, Delgado MJ 2013 Functional analysis of the copy 1 of the *fixNOQP* operon of *Ensifer* *meliloti* under free-living micro-oxic and symbiotic conditions. J Appl Microbiol 114:1772–1781. doi:10.1111/jam.12168. 23414432

[66] Garnerone AM, Cabanes D, Foussard M, Boistard P, Batut J 1999 Inhibition of the FixL sensor kinase by the FixT protein in *Sinorhizobium meliloti*. J Biol Chem 274:32500–32506. doi:10.1074/jbc.274.45.32500. 10542296

[67] Batut J, Daveran-Mingot ML, David M, Jacobs J, Garnerone AM, Kahn D 1989 *fixK*, a gene homologous with *fnr* and *crp* from *Escherichia coli*, regulates nitrogen fixation genes both positively and negatively in *Rhizobium meliloti*. EMBO J 8:1279–1286. 2663474 10.1002/j.1460-2075.1989.tb03502.x PMC400945

[68] Gao M, Nguyen H, Salas González I, Teplitski M 2016 Regulation of *fixLJ* by Hfq controls symbiotically important genes in *Sinorhizobium meliloti*. Mol Plant Microbe Interact 29:844–853. doi:10.1094/MPMI-09-16-0182-R. 27712144

[69] Foussard M, Garnerone AM, Ni F, Soupène E, Boistard P, Batut J 1997 Negative autoregulation of the *Rhizobium meliloti* *fixK* gene is indirect and requires a newly identified regulator, FixT. Mol Microbiol 25:27–37. doi:10.1046/j.1365-2958.1997.4501814.x. 11902723

[70] Zeilstra-Ryalls JH, Gabbert K, Mouncey NJ, Kaplan S, Kranz RG 1997 Analysis of the *fnrL* gene and its function in *Rhodobacter capsulatus*. J Bacteriol 179:7264–7273. doi:10.1128/jb.179.23.7264-7273.1997. 9393689 PMC179675

[71] Poole RK, Anjum MF, Membrillo-Hernández J, Kim SO, Hughes MN, Stewart V 1996 Nitric oxide, nitrite, and Fnr regulation of *hmp* (flavohemoglobin) gene expression in *Escherichia coli* K-12. J Bacteriol 178:5487–5492. doi:10.1128/jb.178.18.5487-5492.1996. 8808940 PMC178372

[72] de Bruijn FJ, Rossbach S, Bruand C, Parrish JR 2006 A highly conserved *Sinorhizobium meliloti* operon is induced microaerobically via the FixLJ system and by nitric oxide (NO) via NnrR. Environ Microbiol 8:1371–1381. doi:10.1111/j.1462-2920.2006.01030.x. 16872401

[73] Müller-Hill B 2006 What is life? The paradigm of DNA and protein cooperation at high local concentrations. Mol Microbiol 60:253–255. doi:10.1111/j.1365-2958.2006.05126.x. 16573677

[74] Müller-Hill B 1998 The function of auxiliary operators. Mol Microbiol 29:13–18. doi:10.1046/j.1365-2958.1998.00870.x. 9701798

[75] Fortino V, Smolander OP, Auvinen P, Tagliaferri R, Greco D 2014 Transcriptome dynamics-based operon prediction in prokaryotes. BMC Bioinformatics 15:145. doi:10.1186/1471-2105-15-145. 24884724 PMC4235196

[76] Bobik C, Meilhoc E, Batut J 2006 FixJ: a major regulator of the oxygen limitation response and late symbiotic functions of *Sinorhizobium meliloti*. J Bacteriol 188:4890–4902. doi:10.1128/JB.00251-06. 16788198 PMC1482993

[77] Levanon SS, San KY, Bennett GN 2005 Effect of oxygen on the *Escherichia coli* ArcA and FNR regulation systems and metabolic responses. Biotechnol Bioeng 89:556–564. doi:10.1002/bit.20381. 15669087

[78] Reyrat JM, David M, Blonski C, Boistard P, Batut J 1993 Oxygen-regulated *in* *vitro* transcription of *Rhizobium meliloti nifA* and *fixK* genes. J Bacteriol 175:6867–6872. doi:10.1128/jb.175.21.6867-6872.1993. 8226629 PMC206811

[79] Malan TP, Kolb A, Buc H, McClure WR 1984 Mechanism of CRP-cAMP activation of *lac* operon transcription initiation activation of the P1 promoter. J Mol Biol 180:881–909. doi:10.1016/0022-2836(84)90262-6. 6098691

[80] Pinedo CA, Gage DJ 2009 HPrK regulates succinate-mediated catabolite repression in the Gram-negative symbiont *Sinorhizobium meliloti*. J Bacteriol 191:298–309. doi:10.1128/JB.01115-08. 18931135 PMC2612420

[81] Garcia PP, Bringhurst RM, Arango Pinedo C, Gage DJ 2010 Characterization of a two-component regulatory system that regulates succinate-mediated catabolite repression in *Sinorhizobium meliloti*. J Bacteriol 192:5725–5735. doi:10.1128/JB.00629-10. 20817764 PMC2953702

[82] Charles TC, Finan TM 1991 Analysis of a 1600-kilobase *Rhizobium meliloti* megaplasmid using defined deletions generated *in vivo*. Genetics 127:5–20. 1849856 10.1093/genetics/127.1.5 PMC1204311

[83] MacLellan SR, Smallbone LA, Sibley CD, Finan TM 2005 The expression of a novel antisense gene mediates incompatibility within the large *repABC* family of α-proteobacterial plasmids. Mol Microbiol 55:611–623. doi:10.1111/j.1365-2958.2004.04412.x. 15659174

[84] Bolger AM, Lohse M, Usadel B 2014 Trimmomatic: a flexible trimmer for Illumina sequence data. Bioinformatics 30:2114–2120. doi:10.1093/bioinformatics/btu170. 24695404 PMC4103590

[85] Trapnell C, Pachter L, Salzberg SL 2009 TopHat: discovering splice junctions with RNA-Seq. Bioinformatics 25:1105–1111. doi:10.1093/bioinformatics/btp120. 19289445 PMC2672628

[86] Trapnell C, Williams BA, Pertea G, Mortazavi A, Kwan G, van Baren MJ, Salzberg SL, Wold BJ, Pachter L 2010 Transcript assembly and quantification by RNA-Seq reveals unannotated transcripts and isoform switching during cell differentiation. Nat Biotechnol 28:511–515. doi:10.1038/nbt.1621. 20436464 PMC3146043

[87] Thomas-Chollier M, Sand O, Turatsinze JV, Janky R, Defrance M, Vervisch E, Brohée S, van Helden J 2008 RSAT: regulatory sequence analysis tools. Nucleic Acids Res 36:W119–W127. doi:10.1093/nar/gkn304. 18495751 PMC2447775

[88] Bailey TL, Williams N, Misleh C, Li WW 2006 MEME: discovering and analyzing DNA and protein sequence motifs. Nucleic Acids Res 34:W369–W373. doi:10.1093/nar/gkl198. 16845028 PMC1538909

[89] Grant CE, Bailey TL, Noble WS 2011 FIMO: scanning for occurrences of a given motif. Bioinformatics 27:1017–1018. doi:10.1093/bioinformatics/btr064. 21330290 PMC3065696

[90] Gupta S, Stamatoyannopoulos JA, Bailey TL, Noble WS 2007 Quantifying similarity between motifs. Genome Biol 8:R24. doi:10.1186/gb-2007-8-2-r24. 17324271 PMC1852410

[91] Meade HM, Long SR, Ruvkun GB, Brown SE, Ausubel FM 1982 Physical and genetic characterization of symbiotic and auxotrophic mutants of *Rhizobium meliloti* induced by transposon Tn*5* mutagenesis. J Bacteriol 149:114–122. 6274841 10.1128/jb.149.1.114-122.1982 PMC216598

[92] Charles TC, Cai GQ, Aneja P 1997 Megaplasmid and chromosomal loci for the PHB degradation pathway in *Rhizobium* (*Sinorhizobium*) *meliloti*. Genetics 146:1211–1220. 9258668 10.1093/genetics/146.4.1211 PMC1208069

[93] Aneja P, Dai M, Lacorre DA, Pillon B, Charles TC 2004 Heterologous complementation of the exopolysaccharide synthesis and carbon utilization phenotypes of *Sinorhizobium meliloti* Rm1021 polyhydroxyalkanoate synthesis mutants. FEMS Microbiol Lett 239:277–283. doi:10.1016/j.femsle.2004.08.045. 15476977

[94] Hanahan D 1983 Studies on transformation of *Escherichia coli* with plasmids. J Mol Biol 166:557–580. doi:10.1016/S0022-2836(83)80284-8. 6345791

[95] Finan TM, Kunkel B, De Vos GF, Signer ER 1986 Second symbiotic megaplasmid in *Rhizobium meliloti* carrying exopolysaccharide and thiamine synthesis genes. J Bacteriol 167:66–72. doi:10.1128/jb.167.1.66-72.1986. 3013840 PMC212841

[96] Schäfer A, Tauch A, Jäger W, Kalinowski J, Thierbach G, Pühler A 1994 Small mobilizable multi-purpose cloning vectors derived from the *Escherichia coli* plasmids pK18 and pK19: selection of defined deletions in the chromosome of *Corynebacterium glutamicum*. Gene 145:69–73. doi:10.1016/0378-1119(94)90324-7. 8045426

