# Supplementary figures and images for: Transcriptome Analysis of Polyhydroxybutyrate Cycle Mutants Reveals Discrete Loci Connecting Nitrogen Utilization and Carbon Storage in Sinorhizobium meliloti
Source: mSystems. 2017 Sep 12;2(5):e00035-17. doi: 10.1128/mSystems.00035-17 (PMC5596199; doi:10.1128/mSystems.00035-17)

pSymA

pSymB

chromosome

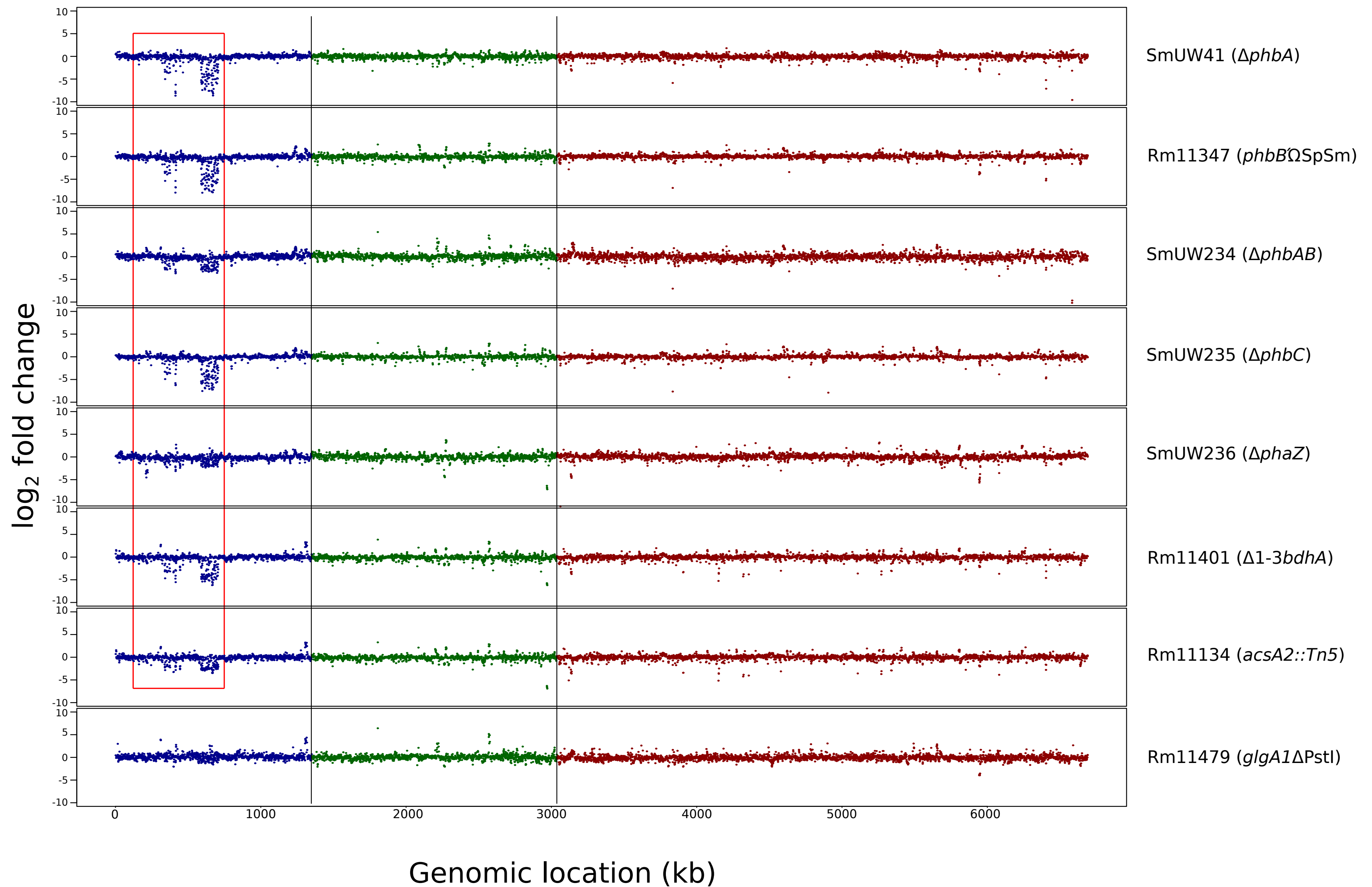

Supplement: FIG S3 [file sys004172129sf4.pdf]

pSymA

pSymB

chromosome

 $\log_2$  fold change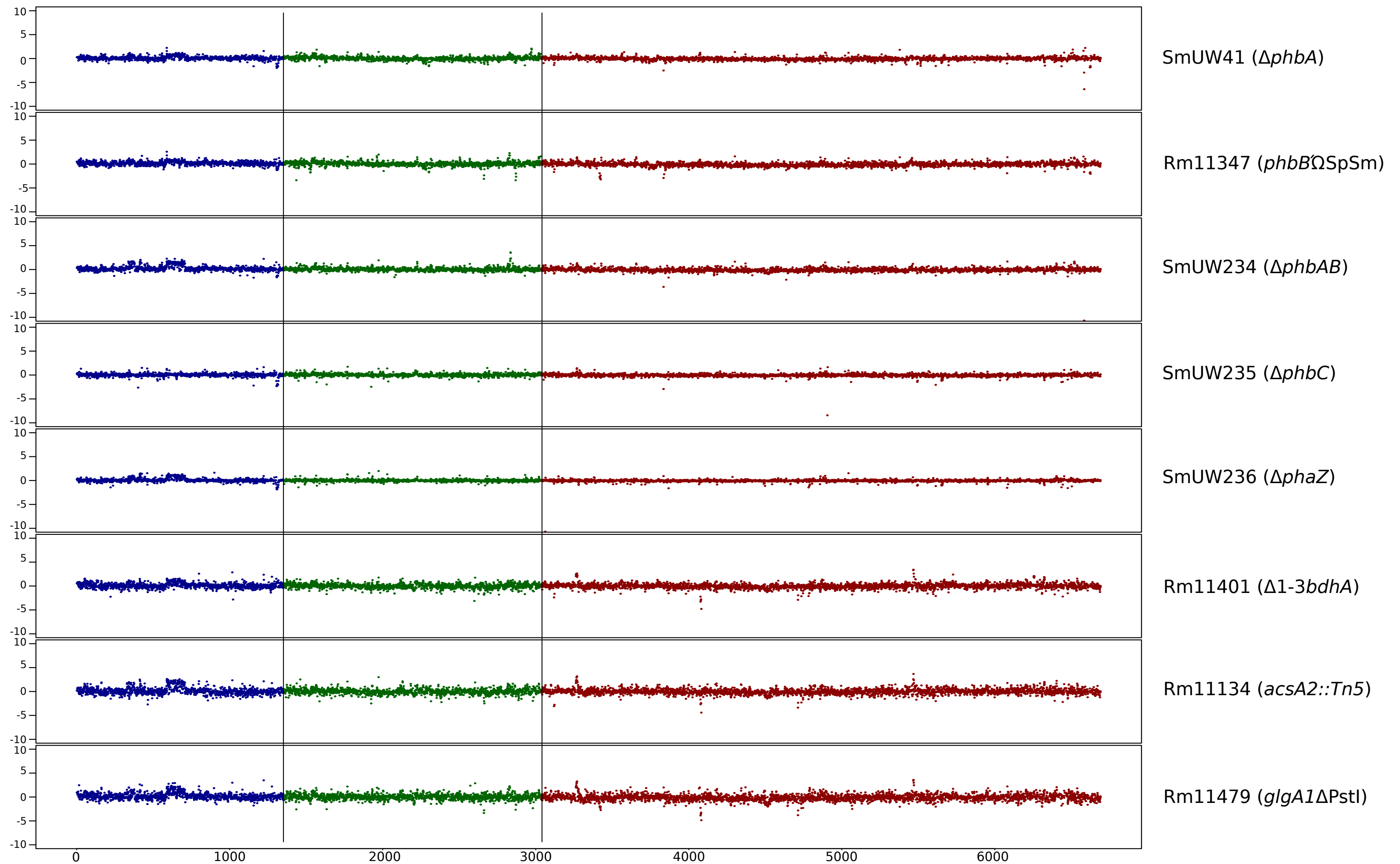

Genomic location (kb)

Supplement: FIG S4 [file sys004172129sf5.pdf]

| cluster <sup>a</sup> | motif                                                                                | e-value | sites <sup>b</sup> | genes in cluster (%) |
|----------------------|--------------------------------------------------------------------------------------|---------|--------------------|----------------------|
| B1                   | 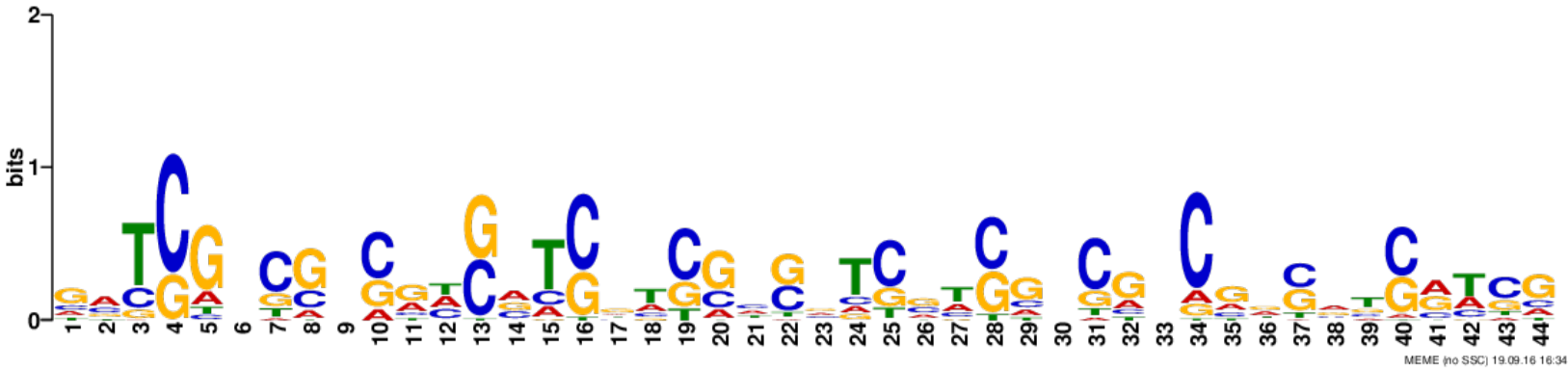    | 4.6e-41 | 163                | 44.4                 |
|                      | 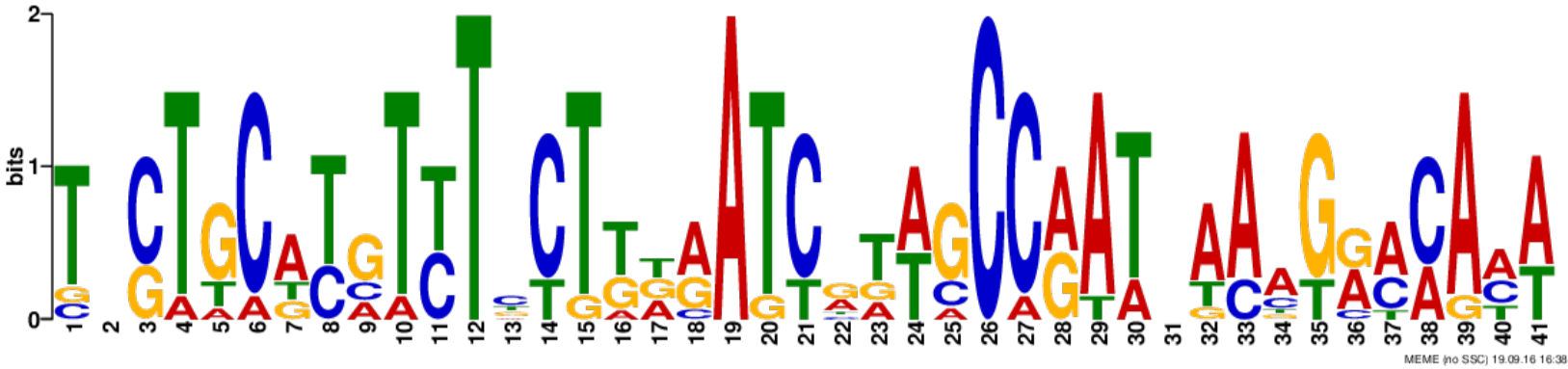    | 5.4e-16 | 9                  | 2.4                  |
| B2                   | 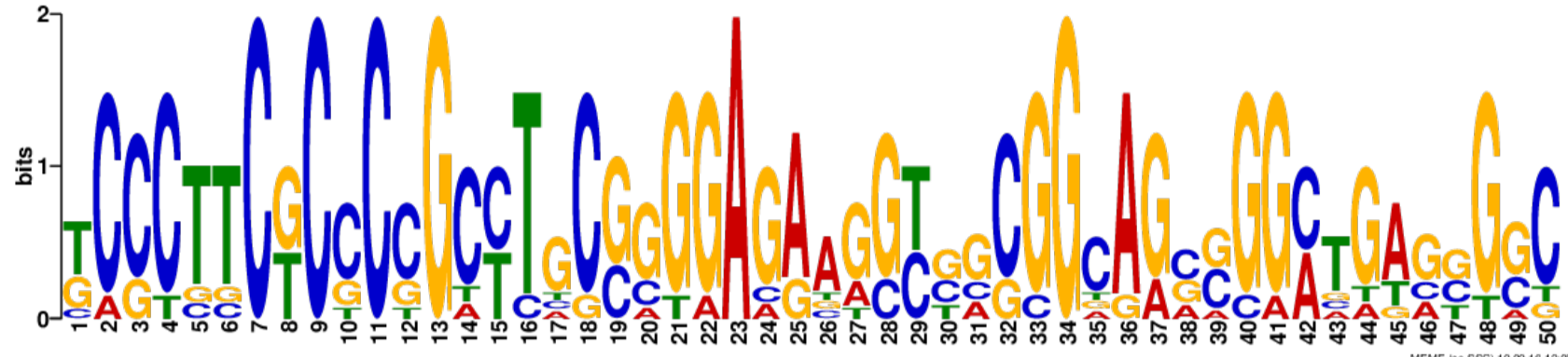    | 6.8e-20 | 9                  | 2.9                  |
|                      | 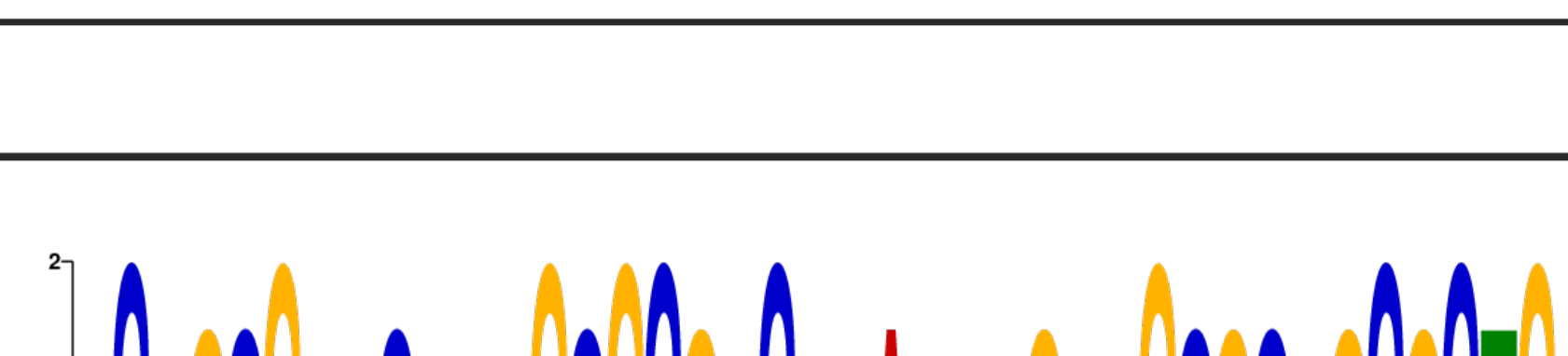   |         |                    |                      |
| B3                   | 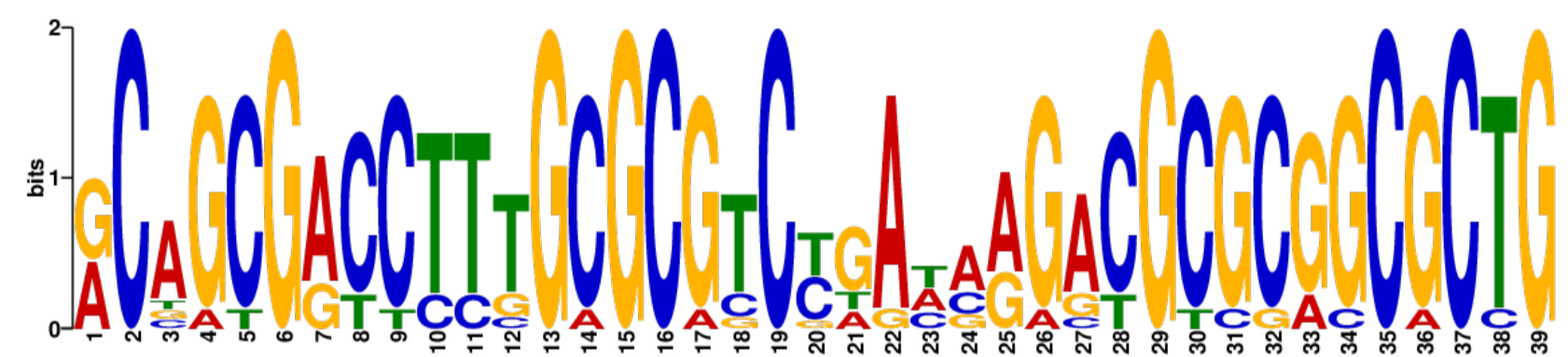  | 1e-51   | 11                 | 1.5                  |
|                      | 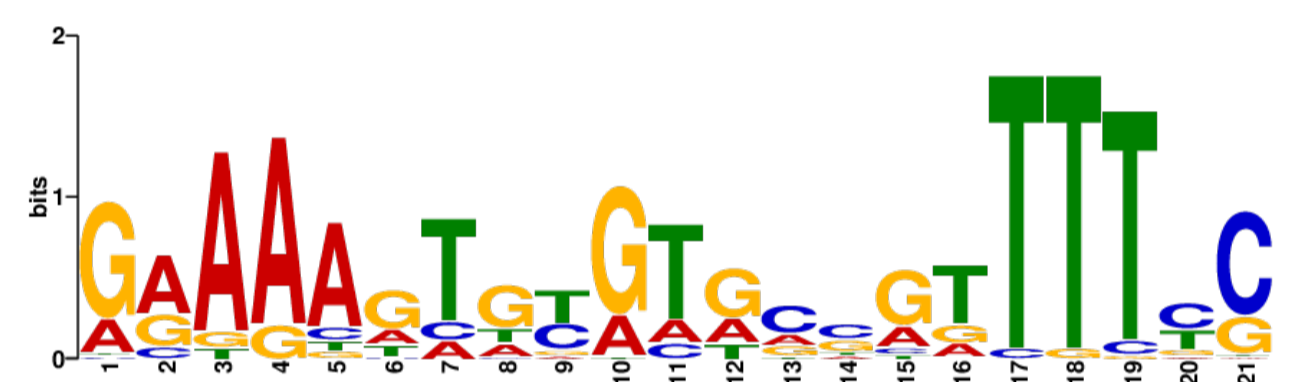  | 9.6e-35 | 51                 | 7.1                  |
|                      | 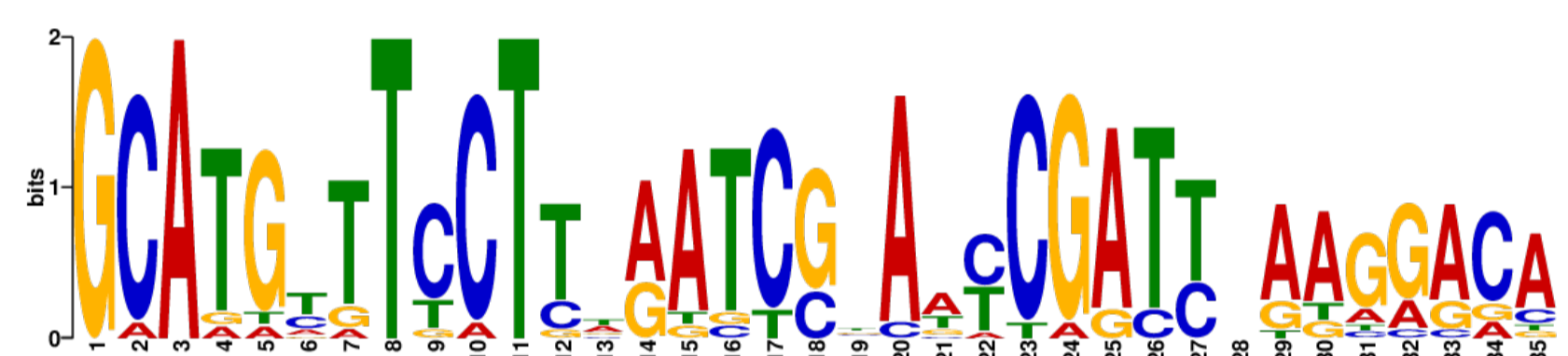  | 2.2e-43 | 14                 | 2.0                  |
|                      | 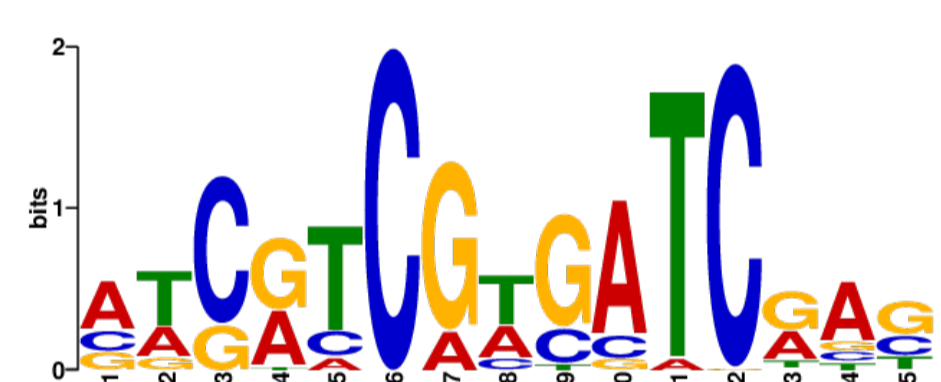  | 1.2e-16 | 84                 | 11.7                 |
| B4B                  | 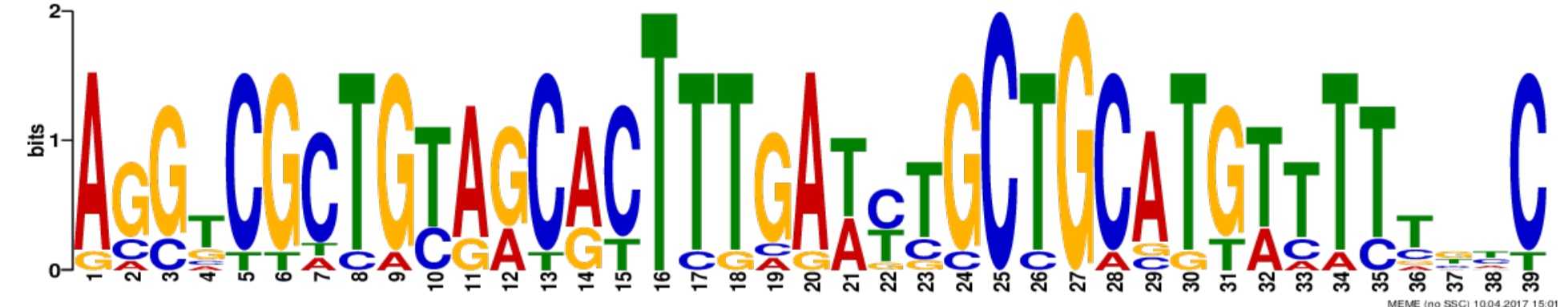 | 1.1e-35 | 10                 | 2.7                  |
|                      | 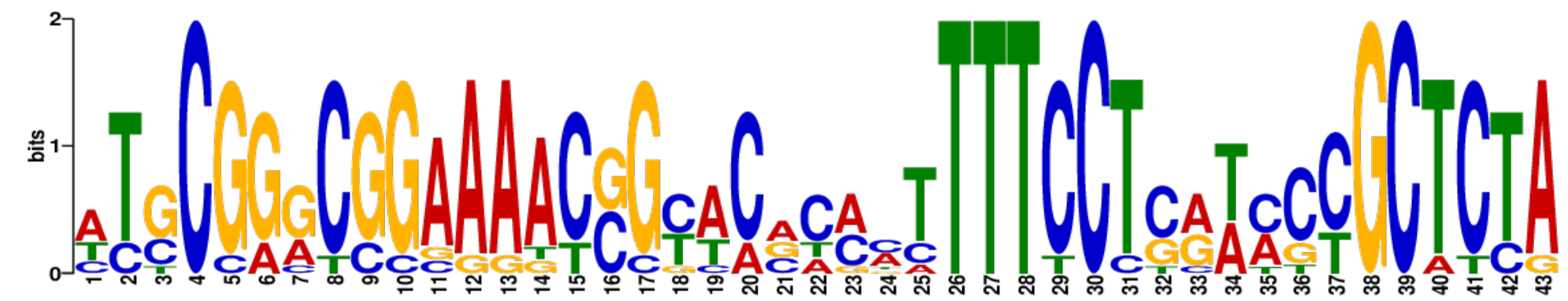 | 3.8e-39 | 10                 | 2.7                  |
|                      | 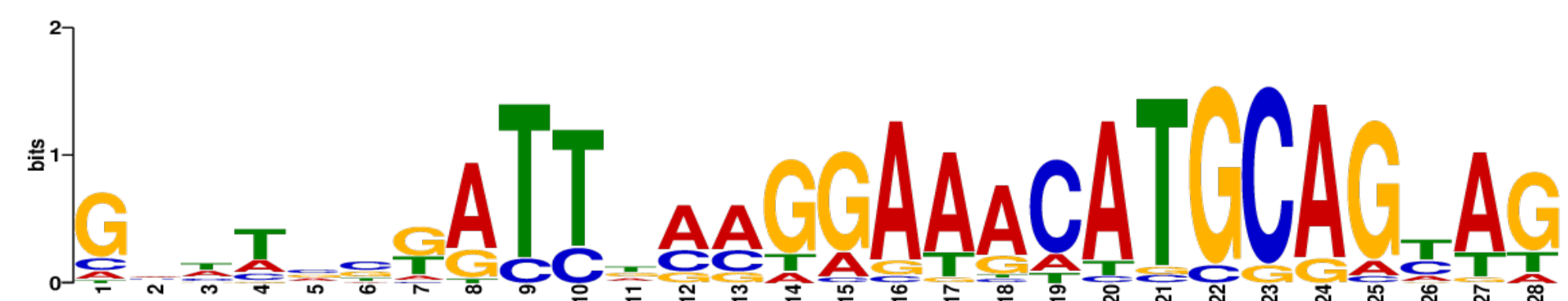 | 9.5e-16 | 21                 | 5.7                  |
| A                    | 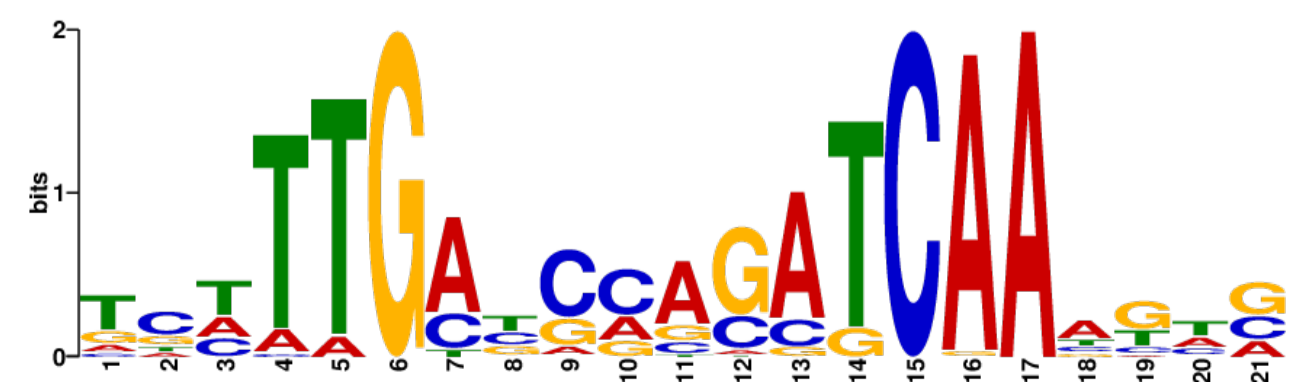  | 3.3e-80 | 47                 | 31.1                 |
|                      | 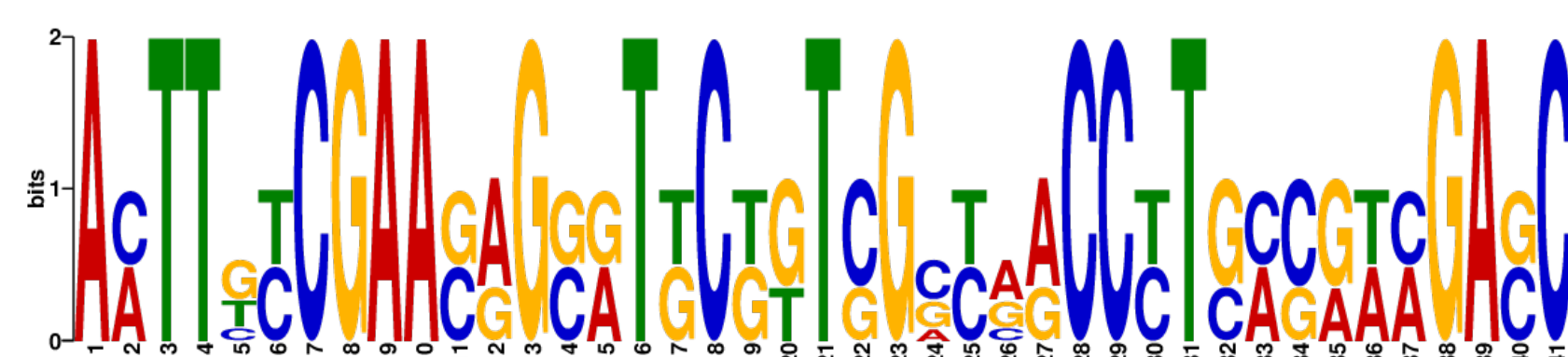  | 5.3e-17 | 6                  | 4.0                  |

Supplement: FIG S6 [file sys004172129sf7.pdf]
