# Supplementary material for: Transcriptome Analysis of Polyhydroxybutyrate Cycle Mutants Reveals Discrete Loci Connecting Nitrogen Utilization and Carbon Storage in Sinorhizobium meliloti
Source: mSystems. 2017 Sep 12;2(5):e00035-17. doi: 10.1128/mSystems.00035-17 (PMC5596199; doi:10.1128/mSystems.00035-17)
Supplement: TABLE S2 [file sys004172129st8.docx]

| Protein | Domain | Domain |
| --- | --- | --- |
| FixK1 | Crp (not in pfam) | cNMP binding |
| SMa0662 | HTH_Crp_2 | cNMP binding |
| SMa1011 | Crp (not in pfam) | cNMP binding |
| SMa1067 | HTH_Crp_2 | cNMP binding |
| SMa1141 | HTH_Crp_2 | cNMP binding |
| SMa1207 | Crp (not in pfam) | cNMP binding |
| FixK2 | HTH_Crp_2 | cNMP binding |
| NnrR | HTH_Crp_2 | cNMP binding |
| SMa1948 | HTH_Crp_2 | cNMP binding |
| SMb21079 | HTH_Crp_2 | cNMP binding |
| SMb21270 | HTH_Crp_2 | cNMP binding |
| SMc01954 | HTH_Crp_2 | cNMP binding |
| SMc02175 | HTH_Crp_2 | cNMP binding |
| SMc03819 | Crp (not in pfam) | cNMP binding |

**Table S2.** A list of all of the Fnr/Crp genes in *S. meliloti* Rm1021 as identified through the presence of two key domains.
